# Evolutionary relationships of ATP-Binding Cassette (ABC) uptake porters

**DOI:** 10.1186/1471-2180-13-98

**Published:** 2013-05-06

**Authors:** Wei Hao Zheng, Åke Västermark, Maksim A Shlykov, Vamsee Reddy, Eric I Sun, Milton H Saier

**Affiliations:** 1 Department of Molecular Biology, University of California at San Diego, La Jolla, CA, 92093-0116, USA; 2 Present address: Medical College of Wisconsin, Milwaukee, WI, USA; 3 Present address: University of Michigan Medical School, Ann Arbor, MI, USA; 4 Present address: University of Calgary, Calgary, AB, Canada; 5 Present address: Burnham Institute, La Jolla, CA, USA

**Keywords:** ATP-Binding Cassette, ABC, Uptake, Transport proteins, Membranes, Protein superfamilies, Comparisons

## Abstract

**Background:**

The ATP-Binding Cassette (ABC) functional superfamily includes integral transmembrane exporters that have evolved three times independently, forming three families termed ABC1, ABC2 and ABC3, upon which monophyletic ATPases have been superimposed for energy-coupling purposes [e.g., J Membr Biol 231(1):1-10, 2009]. The goal of the work reported in this communication was to understand how the integral membrane constituents of ABC uptake transporters with different numbers of predicted or established transmembrane segments (TMSs) evolved. In a few cases, high resolution 3-dimensional structures were available, and in these cases, their structures plus primary sequence analyses allowed us to predict evolutionary pathways of origin.

**Results:**

All of the 35 currently recognized families of ABC uptake proteins except for one (family 21) were shown to be homologous using quantitative statistical methods. These methods involved using established programs that compare native protein sequences with each other, after having compared each sequence with thousands of its own shuffled sequences, to gain evidence for homology. Topological analyses suggested that these porters contain numbers of TMSs ranging from four or five to twenty. Intragenic duplication events occurred multiple times during the evolution of these porters. They originated from a simple primordial protein containing 3 TMSs which duplicated to 6 TMSs, and then produced porters of the various topologies via insertions, deletions and further duplications. Except for family 21 which proved to be related to ABC1 exporters, they are all related to members of the previously identified ABC2 exporter family. Duplications that occurred in addition to the primordial 3 → 6 duplication included 5 → 10, 6 → 12 and 10 → 20 TMSs. In one case, protein topologies were uncertain as different programs gave discrepant predictions. It could not be concluded with certainty whether a 4 TMS ancestral protein or a 5 TMS ancestral protein duplicated to give an 8 or a 10 TMS protein. Evidence is presented suggesting but not proving that the 2TMS repeat unit in ABC1 porters derived from the two central TMSs of ABC2 porters. These results provide structural information and plausible evolutionary pathways for the appearance of most integral membrane constituents of ABC uptake transport systems.

**Conclusions:**

Almost all integral membrane uptake porters of the ABC superfamily belong to the ABC2 family, previously established for exporters. Most of these proteins can have 5, 6, 10, 12 or 20 TMSs per polypeptide chain. Evolutionary pathways for their appearance are proposed.

## Background

Over the past twenty-five years, more than six hundred families of transport systems have been identified, and these are presented in the Transporter Classification Database, TCDB (http://www.tcdb.org). Classification is based on the transmembrane constituents that shape the membrane channels, rather than co-functioning auxiliary proteins including the energy coupling constituents [2-4]. Among the many protein families found in this database is the ATP-binding cassette (ABC) superfamily (TC# 3.A.1), the largest functional superfamily of primary active transporters found in nature. Many of these systems have been functionally characterized, and high resolution 3-dimensional structures are available for a few of them.

The ABC functional superfamily consists of both uptake and efflux transport systems, all of which have been shown to utilize ATP hydrolysis to energize transport [5]. The X-ray crystallographic structures of several uptake porters have been solved [6,7]. In general, individual porters of the ABC superfamily contain integral membrane domains or subunits and cytoplasmic ATP-hydrolyzing domains or subunits. Unlike the efflux porters, many uptake systems additionally possess extracytoplasmic solute-binding receptors, assisting in the high affinity transport of solutes across the membrane [8,9]. Some ABC uptake systems lack these receptors, and this ABC subsuperfamily has been referred to as the ECF subsuperfamily of the ABC functional superfamily [10,11] (EI Sun and MH Saier, manuscript in press).

ABC exporters are polyphyletic, meaning that they have arisen through multiple independent pathways to yield distinctive protein families [1]. In fact, they have arisen at least three times independently, following three different pathways. The members of any one of these three families are demonstrably homologous to one another, but homology could not been established when comparing members of one family with those of another. ABC1 exporters arose by intragenic triplication of a primordial genetic element encoding a two-transmembrane segment (TMS) hairpin structure, yielding six TMS proteins. ABC2 transporters arose by intragenic duplication of a primordial genetic element encoding three TMSs, again yielding 6 TMS proteins. ABC3 porters arose with or without duplication of a primordial genetic element encoding four TMSs, resulting in proteins having four, eight, or ten TMSs [1,12]. Only in this last mentioned family are the unduplicated 4 TMS proteins found in present day porters, and they are in the membrane as pairs, forming hetero- or homo-dimers [12]. Because of the limited organismal distribution and minimal sequence divergence between the protein members and the repeat units in the ABC3 family, this last family is believed to have evolved most recently [1,12]. It seems likely that the ABC2 family arose first, that the ABC1 family arose next, and that the ABC3 family arose last [1].

In this study we predict the evolutionary pathways by which ABC uptake systems of differing topologies appeared. With several technological improvements, this has become feasible, first, because of the availability of more sensitive software [13-16], second, because of the availability of larger numbers of homologues resulting from genome sequencing, and third, because of the application of the Superfamily Principle [17,18]. This principle simply states that if protein A is homologous to protein B, and protein B is homologous to protein C, then protein A must be homologous to protein C, regardless of whether significant sequence similarity can be documented for proteins A and C. Homology by definition means derived from a common ancestral protein. It is thus unnecessary to identify regions of high sequence similarity between two proteins if one or more sequences of adequate sequence similarity can be found that interlinks the aforementioned two sequences.

To establish homology between repeat elements in the transmembrane domains of ABC importers, we used the Superfamily Principle as defined above to extend the significant internal homology decisions to other evolutionarily related proteins (e.g., derived from a common ancestor) [17,18]. This principle has been used to establish homology for distantly related members of extensive superfamilies [13,19-21]. As documented in this communication, we have used statistical means to establish homology for all ABC uptake transporters except for TC family 3.A.1.21 which clearly belongs to the ABC1 family. Additionally, we have established homology for internal repeat elements in representative transmembrane domains [4,17,18]. Finally, we have obtained preliminary evidence that two of the six primordial TMSs in ABC2 protein (TMSs 3 and 4) gave rise to the 2 TMS repeat elements in ABC1 porters, suggesting that the evolution of ABC2 porters preceeded that of ABC1 porters.

Many families of integral membrane transport proteins evolved independently of each other following different evolutionary pathways [19]. These pathways involved intragenic multiplication events where the primordial genes presumably encoded channel-forming peptides, usually with one, two or three α-helical TMSs [19]. They duplicated, triplicated or quadruplicated—sometimes in a single step, sometimes in more than one step [19,22,23].

The bacterial maltose transport system proteins, MalF (P02916) and MalG (P68183) are two distinct membrane proteins that together comprise the channel of an ABC superfamily member. High resolution structural information is available for this system (TC# 3.A.1.1.1). Consequently, it is known that these two proteins differ in their TMS architecture. MalF has a 3 + 5 TMS structure whereas MalG has a 3 + 3 TMS structure. We here propose that these proteins, and almost all integral membrane constituents of ABC uptake systems, are of the ABC2-type as noted above, arising from a 3 + 3 repeat topology. This raises the question of how the MalF protein arose from a MalG-like precursor. The MalF protein contains a long hydrophilic sequence insert between TMS 3 and TMS 4. Since ABC2 proteins resulted from a 3 TMS domain duplication, the question remained which of the MalF TMSs are the extra ones. Since MalF and MalG are structurally determined membrane proteins, it was possible to draw conclusions from the publicly available coordinate sets in the Protein Data Bank (PDB), for example, from chains F and G in “2R6G” from *E. coli* K12. We provide evidence that the extra 2 TMSs in MalF relative to MalG are TMSs 1 and 2.

The results reported here strongly suggest that the membrane constituents of ABC uptake transporters evolved through pathways starting with a primordial 6 TMS ABC2 porter. Multiple and pairwise alignments as well as hydropathy plots were created and analyzed to elucidate the evolutionary appearance of this topologically diverse group of ABC uptake porters. The two primary structural repeat elements have 5 or 6 TMSs which duplicated in many such proteins and quadruplicated in a few. Although some uncertainty exists regarding the precise topologies of some of these integral membrane proteins, we could document their internal duplications and propose the routes taken during their evolutionary histories.

## Results

### Demonstration that most ABC uptake transporters are homologous

The aim of this section is to establish common origins for the integral membrane constituents of most ABC uptake systems. Initially, the integral membrane constituents of one uptake transporter from each family was blasted using the BLAST search tool in TCDB (TC-BLAST). The resulting proteins were examined, and those that belonged to uptake systems with e-values of smaller than 1e^-4^ were retained for further studies. An example of the BLAST output is shown in Additional file 1: Table S1 where the query sequence was MalF of *E. coli* (TC# 3.A.1.1.1).

Using the Multiple Sequence Alignment Program with Displayed TMSs (MAP-TMS) from TCDB (http://www.tcdb.org), the query sequence and the output sequences were aligned, and their transmembrane regions were predicted. If more than 60 residues containing the corresponding transmembrane α-helical segments (TMSs) aligned between two proteins, and they gave an e-value of 10^-7^ or smaller, they were considered homologous. If the e-value was greater than 10^-7^, we compared both sequences using the GAP program. By our criteria, a comparison score of ≥ 10 standard deviations (S.D.), as defined by the GAP program, indicates that the two sequences are homologous (see Methods). For instance, the sequences YfeC (TC# 3.A.1.15.4) and FhuB (TC# 3.A.1.14.3) were compared using the GAP program, and the comparison score (quality subtracted from average quality divided by the program’s S.D. value) computed was 18 S.D., well-above the value of 10 S.D. needed to establish homology (Additional file 1: Figure S1).

Using TC BLAST, the Multiple Sequence Alignment Program with Displayed TMSs, GAP and the Superfamily Principle, we could show that large portions of representative transporters from each of the various families of uptake porters are homologous (Table 1). Initially, transporters of some families could not be shown to be homologous using these methods. Membrane proteins from these subfamilies were then blasted against the NCBI protein databank, and the gi numbers of hits were obtained using gi-Extract from TCDB. The gi numbers of the protein homologues were searched on NCBI in order to obtain their FASTA sequences, and a modified CD-Hit program was used to eliminate redundant and closely related proteins [13,24]. Protein homologues from different transporters were compared using SSearch. Comparison scores above 10 S.D. were sought. A combination of programs such as GAP and the Global Alignment Program With Displayed TMSs (GAP-TMS) (http://www.tcdb.org) were used to establish homology. Table 1 presents evidence that by the criteria presented here and in our previous publications, all integral membrane constituents of ABC uptake porters except TC family 3.A.1.21 are homologous (see Methods).

**Table 1:** **Demonstration that most ABC uptake membrane proteins are homologous**^**1,2,3**^

	**1.1 MalG**	**2.1 RbsC**	**2.4 XylH**	**3.2 GlnP**	**3.8 AapM**	**4.1 LivM**	**12.3 OPBD**	**12.8 OpuBB**	**14.3 FhuB**	**14.16 FeuC**	**20.1 BitE**	**23.2 CbiQ**	**25.1 BioN**	**26.1 CbiQ**	**28.1 QrtT**	**29.1 MtsU**
1.6 CymF											12					
2.5 GguB										**13SD**						
2.10 PnrE				**16SD**												
3.2 GlnP							**15SD**									
3.19 GtsC	16															
4.4 UrtB			**14SD**													
5.2 DppC							**13.5SD**									
6.3 CysW											14					
6.5 WtpB				8												
7.1 PstA							**12SD**									
8.1 ModB	10															
9.2 PhnE								12								
10.3 FbpB											21					
11.4 ChtK	8															
13.1 BtuC									30							
15.4 YfeC									**18SD**							
16.3 CmpB							10									
17.2 SsuC							9									
18.1 CbiQ												**15SD**				
19.1 ThiP											**17SD**					
22.1 CbiQ														**13SD**		
24.1 MetI							9									
25.1 BioY homologue gi145224049				**11SD**	**11SD**											
26.7 EcfT													8			
27.2 Tgd1 homologue gi54023080						**11SD**										
28.1 QrtT														13		
29.1 MtsU												6				
30.1 YkoC														7	**17SD**	
31.1 HtsTUV														**14SD**		
32.1 CbrT																**18.9SD**
33.1 MtaT															6	13
34.1 TrpY		12														

^1^ Since completion of the work reported here, a new ABC family (3.A.1.35; CPC) has been introduced into TCDB. 35.1; EtcT gave e^-12^ with 26.5 and e^-9^ with 30.1 and 33.1, thus indicating homology between families 26, 30, 33 and 35.

^2^ Usually, superfamilies in TCDB, half of which have been introduced during the last 2.5 years, contain multiple TC families (and are hence, by definition, more divergent in sequence than the APC family 2.A.3). However, in 2.A.3, all recognized members of this family were initially included under 2.A.3. This is a historical fact that cannot be readily corrected because the IUBMB and UniProt require a stable system of classification. Subsequently, we could show that other families previously existing in TCDB were members of this superfamily. The same was true for the MFS. Thus, we call what would normally be called “subfamilies” the families for both the MFS (2.A.1) and the APC (2.A.3). The same is true for the ABC functional superfamily, except that the membrane proteins actually comprise three superfamilies, ABC1, ABC2 and ABC3 as discussed above [16].

^3^ The numbers in bold indicate comparison scores expressed in S.D [16]. Non-bolded numbers are the exponential numbers (e-values) obtained with TC-BLAST. For instance, the number “12” in the first row of column 12 indicates that the comparison score between 1.6 CymF and 20.1 BitE was e^-12^. The TC# provided is the family/protein number (e.g. 1.1 for MalF and MalG, the two membrane constituents of the *E. coli* maltose transporter). The first three digits in the TC# (3.A.1.) refer to the ABC functional superfamily and are not shown. They are the same for all entries. The protein TC# is followed by the protein abbreviation. All members of a single family are demonstrably homologous, giving high comparison scores (greater than 15 S.D.). Any two families for which a number is provided in the table below are demonstrably homologous based on the criteria stated in the Methods section. All proteins are within the ABC superfamily (3.A.1), but only the family and protein TC#s are provided below, e.g. 1.6 means 3.A.1.1.6, i.e., ABC family 1, member 6.

### Topological analyses of ABC uptake system

ABC uptake systems, found only in prokaryotes and chloroplasts, contain porters of diverse topological types, and in this section we attempt to predict these topologies. Our studies, reported below, allow us to propose that the primordial transporter contained three TMSs, which duplicated internally to give six TMS homologues [1]. As demonstrated here, membrane constituents of ABC uptake systems except those of family 21 are of the ABC2 type. However, the actual transporters appearing on the TCDB website contain various numbers of TMSs that range from four or five to twenty. For some families of uptake systems such as families 1, 3 and 14, the porters are more topologically diverse than those from other families such as 8, 11 and 17. Table 2 presents these families and summarizes the topological types predicted for members of uptake porter families.

**Table 2:** **Predicted topologies for members of the 34 families of uptake porters in the ABC superfamily (TC# 3.A.1)**^**1**^

	**Family name**	**No. of membrane proteins in TCDB**	**No. of membrane proteins/system**	**Average predicted #TMSs**	No. of predicted TMSs for family members. The “*” indicates the most abundant topological type	**Predicted number of TMS (# of such proteins is shown in parentheses)**
1	**The Carbohydrate Uptake Transporter-1 (CUT1) Family**	35	2	6.6 ± 1.1	6*,7, 8	6 (16), 7 (8), 8 (10)
2	**The Carbohydrate Uptake Transporter-2 (CUT2) Family**	17	1 or 2	9.4 ± 1.1	7, 8, 9, 10*, 12	7 (1), 8 (2), 9 (5), 10 (8), 12 (1)
3	**The Polar Amino Acid Uptake Transporter (PAAT) Family**	21	2 or 1	5.9 ± 1.8	5*, 6, 8, 9, 10, 11	5 (15), 6 (2), 9 (1), 10 (1), 11 (1)
4	**The Hydrophobic Amino Acid Uptake Transporter (HAAT) Family**	6	2	9.8 ± 0.7	9, 10*, 11	9 (2), 10 (3), 11 (1)
5	**The Peptide/Opine/Nickel Uptake Transporter (PepT) Family**	27	2	6.2 ± 1.2	5, 6*, 7, 8	6 (19), 7 (3), 8 (2)
6	**The Sulfate/Tungstate Uptake Transporter (SulT) Family**	7	1 or 2	5.7 ± 0.5	5, 6*	5 (2), 6 (5)
7	**The Phosphate Uptake Transporter (PhoT) Family**	2	2	6.5 ± 0.5	6*, 7	6 (1), 7 (1)
8	**The Molybdate Uptake Transporter (MolT) Family**	2	1	5.0 ± 0	5	5 (2)
9	**The Phosphonate Uptake Transporter (PhnT) Family**	2	1	9.0 ± 3.0	6*, 12*	6 (1), 12 (1)
10	**The Ferric Iron Uptake Transporter (FeT) Family**	4	1	11.8 ± 0.4	12	11 (1), 12 (3)
11	**The Polyamine/Opine/Phosphonate Uptake Transporter (POPT) Family**	6	2	6.0 ± 0	6	6 (6)
12	**The Quaternary Amine Uptake Transporter (QAT) Family**	13	1 or 2	6.4 ± 1.3	5*, 6, 7, 8, 9	5 (4), 6 (4), 7 (2), 8 (2), 9 (1)
13	**The Vitamin B12 Uptake Transporter (B12T) Family**	1	1	9.0 ± 0	9*	9 (1)
14	**The Iron Chelate Uptake Transporter (FeCT) Family**	27	2 or 1	9.6 ± 3.9	7, 8, 9*, 10, 11, 20	7 (3), 8 (1), 9 (10), 10 (4), 11 (1), 20 (2)
15	**The Manganese/Zinc/Iron Chelate Uptake Transporter (MZT) Family**	11	1 or 2	8.0 ± 0.9	7, 8*, 9	7 (4), 8 (3), 9 (4)
16	**The Nitrate/Nitrite/Cyanate Uptake Transporter (NitT) Family**	3	1	6.0 ± 0	6	6 (3)
17	**The Taurine Uptake Transporter (TauT) Family**	6	1	6.0 ± 0	6	6 (6)
18	**The Cobalt Uptake Transporter (CoT) Family**	1	2 (ECF)	6.0 ± 0	5*, 6*	6 (1)
19	**The Thiamin Uptake Transporter (ThiT) Family**	2	1	12.0 ± 0	12*	12 (2)
20	**The *Brachyspira *Iron Transporter (BIT) Family**	1	2	7.0 ± 0	6, 7	7 (1)
21 (ABC1)	**The Siderophore-Fe3+ Uptake Transporter (SIUT) Family**	2	2 (ECF)	6.5 ± 0.5	6, 7	6 (1), 7 (1)
22	**The Nickel Uptake Transporter (NiT) Family**	1	2 (ECF)	5.0 ± 0	5	5 (1)
23	**The Nickel/Cobalt Uptake Transporter (NiCoT) Family**	2	2 (ECF)	1.5 ± 0.5	5, 6*, 7	6 (1), 7 (1)
24	**The Methionine Uptake Transporter (MUT) Family**	4	1	5.0 ± 0	5	5 (4)
25	**The Biotin Uptake Transporter (BioMNY) Family**	1	2 (ECF)	5.0 ± 0	5*	5 (1)
26	**The Putative Thiamine Uptake Transporter (ThiW) Family**	7	2 (ECF)	5.6 ± 0.7	5	5 (4), 6 (2), 7 (1),
27	**The γ-Hexachlorocyclohexane (HCH) Family**	5	1	5.4 ± 0.5	5*, 6	5 (3), 6 (2)
28	The Queusine (Quesusine) Family	2	2 (ECF)	5.5 ± 0.5	5, 6	5 (1), 6 (1)
29	The Methionine precursor (Met-P) Family	2	2 (ECF)	5.5 ± 0.5	5, 6	5 (1), 6 (1)
30	The Thiamin precursor (Thi-P) Family	2	2 (ECF)	6.0 ± 0	4, 6	6 (2)
31	**The Unknown-ABC1 (U-ABC1) Family**	2	2 (ECF)	6.0 ± 0	6	6 (2)
32	The Cobalamine Precursor (B_12_-P) Family	2	2 (ECF)	8.0 ± 0	6, 8	6 (1), 8 (1)
33	The Methylthioadenosine (MTA) Family	2	2	6.5 ± 0	6, 7	6 (1), 7 (1)
34	The Tryptophan (TrpXYZ) Family	1	1	8.0 ± 0	8	8 (1)
35	The Cobalamin precursor/Cobalt (CPC) Family	2	2	5.7 ± 1	6	4 (2) 6 (2) 7 (2)

^1^ Most uptake porters are of the ABC2 type. However, TC# 3.A.1.21 porters belong to the ABC1 type. Blasting family 21 porters yielded ABC1 exporters in families TC# 3.A.1.101 to TC# 3.A.1.113 [9].

Proteins were derived from TCDB.

### Identifying internal repeats

#### Internal 3 TMS repeats in 6 TMS proteins

**Figure 1:**
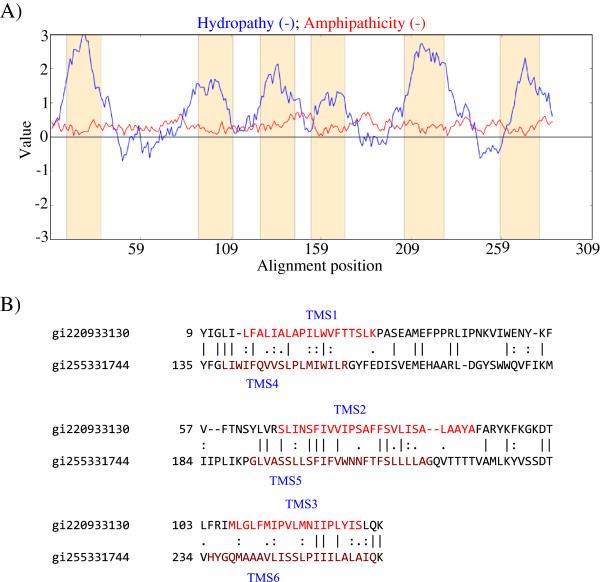
**Internal 3 TMS repeats in 6 TMS proteins. A** (left). Hydropathy plot of MalG (TC# 3.A.1.1.1), a six TMS membrane porter. Blue lines denote Hydropathy; Red lines denote Amphipathicity; Orange bars mark transmembrane segments as predicted by HMMTOP. **B** (right). TMSs 1–3 of gi220933130 aligning with TMSs 4–6 of gi255331744 yielded a comparison score of 10.9 S.D. with 40.3% similarity and 27.7% identity. The numbers at the beginning of each line refer to the residue numbers in each of the proteins. TMSs are indicated in red lettering. Vertical lines indicate identities; colons indicate close similarities, and periods indicate more distant similarities.

As previously shown for ABC2 exporters, we here show that membrane proteins of ABC uptake porters arose by an initial gene duplication event where a 3 TMS-encoding genetic element duplicated to give 6 TMS proteins. Initial sequences were obtained from TCDB using MalG from *E. coli* (TC# 3.A.1.1.1) as the query sequence in BLAST searches of the NCBI databank. The crystallographic structure of the *E. coli* maltose transporter has been solved [7], and MalG has six TMSs, in agreement with the topological predictions obtained by the WHAT, HMMTOP and TMHMM 2.0 programs. Figure 1A shows a hydropathy plot of MalG obtained with the WHAT program [25].

The N-terminal half of MalG, containing TMSs 1–3, was compared with TMSs 4–6 using the GAP program. The resulting comparison score, expressed in S.D., was below 10 and therefore did not prove the presence of an internal repeat. Homologues of MalG were obtained by using the NCBI BLAST, SSearch and gi-Extract programs. The redundant and very similar homologues were eliminated using the CD-Hit program with a cut-off value of 90% identity, and fragmentary sequences were manually eliminated. The rest of the homologues were aligned using ClustalX, and their TMS positions were located in the resulting alignment file. Search was then used to compare the first three TMSs of all homologues against their second three TMSs. The results were transferred to the computer by the program Fugu. When viewing a pair of sequences giving a high comparison score, the GAP and MAP-TMS programs from TCDB were used to confirm that the TMSs of homologues matched with TMSs in MalG. All of these alignments yielded comparison scores well above 10 standard deviations, between MalG and its homologues. For example, a homologue of MalG with gi number 255331744 gave a value of 43 S.D. with 46% similarity and 31% identity when compared with the *E. coli* MalG (Additional file 1: Figure S2A). In fact, many MalG homologues proved to be homologous throughout their lengths with all six TMSs aligning well with each other.

An alignment between gi220933130 and MalG is shown in Additional file 1: Figure S2B, demonstrating that all of their TMSs align. The alignment of these two sequences gave a comparison score of 48 S.D. with 46.8% similarity and 37.1% identity. These results demonstrate that all members of family 3.A.1.1 are homologous throughout their lengths. Therefore, it is appropriate to compare TMSs 1–3 with TMSs 4–6 with each other for any of these homologs.

Having established that all TMSs among the proteins that will be examined to prove homology between the two halves paired up and gave highly significant comparison scores, the next step was to determine if MalG homologues contain internal repeats. The comparison in Figure 1B shows TMSs 1–3 of gi220933130 aligning with TMSs 4–6 of gi255331744. This resulted in a comparison score of 10.9 S.D., thereby establishing that TMSs 1–3 are homologous to TMSs 4–6. Similar procedures were used in the analyses reported below.

#### Internal six TMS repeats in twelve TMS proteins

**Figure 2:**
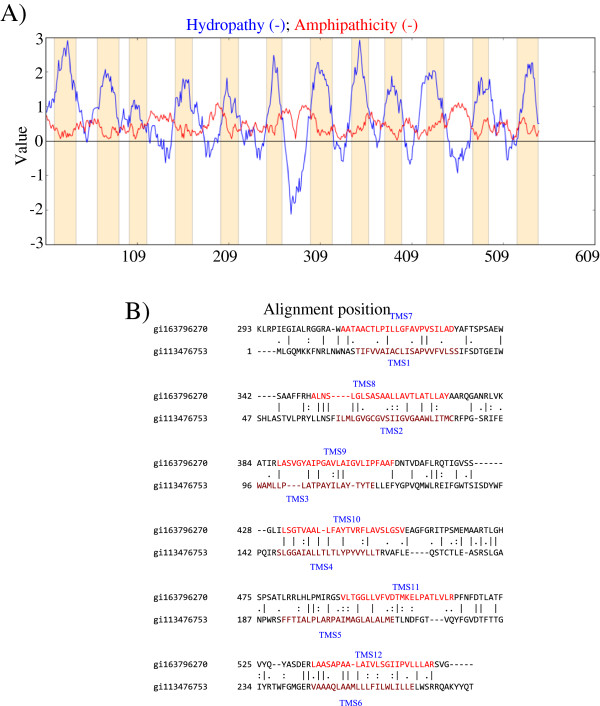
**Internal 6 TMS repeats in 12 TMS proteins. A** (left). Hydropathy plot of the ferric iron porter, FutB. Blue lines denote hydropathy; Red lines denote amphipathicity; Orange bars mark transmembrane segments as predicted by HMMTOP. **B** (right). TMSs 7– 12 of gi163796270 aligned with TMSs 1–6 of gi113476753, yielding a comparison score of 13.7 S.D. with 36.3% similarity and 27.1% identity. The numbers at the beginning of each line refer to the residue numbers in each of the proteins. TMSs are indicated in red lettering. Vertical lines indicate identities; colons indicate close similarities, and periods indicate more distant similarities.

In some instances, six TMS transporters duplicated to produce proteins with twelve TMSs, and in this section, such duplications are demonstrated. A representative twelve TMS protein found in TCDB is the ferric iron porter FutB (TC# 3.A.1.10.2), many homologues of which are present in cyanobacteria (Figure 2A).

Two twelve TMS homologues are gi113476753 and gi163796270. By using GAP-TMS (http://www.tcdb.org), we showed that their TMSs aligned with FutB. The alignment between the established ferric iron porter and gi113476753 is shown in Additional file 1: Figure S3A. As indicated by the GAP program, the comparison score calculated for this alignment was 305 S.D. (67.5% similarity and 59.6% identity).

The TMS alignment between the ferric iron transporter and gi163796270 is shown in Additional file 1: Figure S3B. It is clear that TMSs 1–12 of the homologue pairs up with the corresponding TMSs in FutB. The GAP program yielded a comparison score of 188 S.D. (57.7% similarity, 49.5% identity).

The first six TMSs of gi113476753 were aligned with the second six TMSs of gi163796270 (Figure 2B). The GAP program gave a comparison score of 13.7 S.D. with 36.3% similarity and 27.1% identity, showing that the two sequences are homologous.

#### Internal five TMS repeats in some 10 TMS transporters

**Figure 3:**
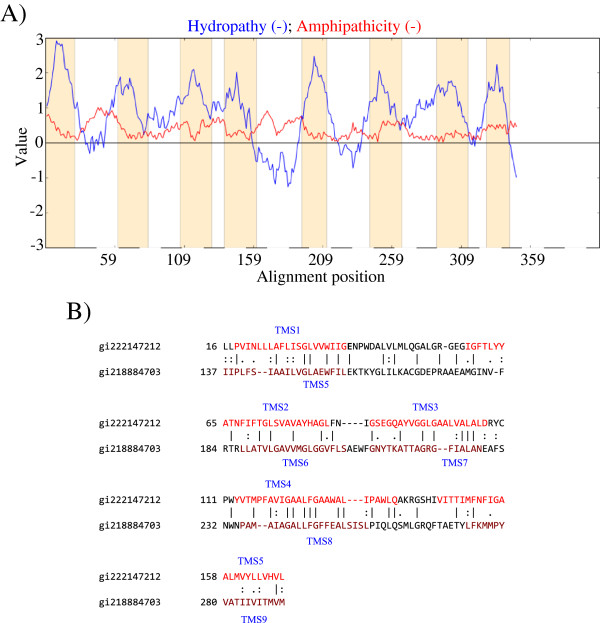
**Internal 5 TMS repeats in some 10 TMS transporters. A** (left). Hydropathy plot of RnsC (TC# 3.A.1.2.12). Blue lines denote Hydropathy; Red lines denote Amphipathicity; Orange bars mark transmembrane segments as predicted by HMMTOP. **B** (right). Putative TMSs 1– 5 of gi222147212 are aligned with putative TMSs 6–10 of gi218884703, yielding a comparison score of 14.9 S.D. with 41.1% similarity and 29.5% identity. The numbers at the beginning of each line refer to the residue numbers in each of the proteins. TMSs are indicated in red lettering. Vertical lines indicate identities; colons indicate close similarities, and periods indicate more distant similarities.

In this section, some 10 TMS proteins are shown to have arisen by duplication of a 5 TMS element. A representative putative ten TMS uptake porter, RnsC (TC# 3.A.1.2.12) and its close homologues, usually predicted to have a 10 TMS topology using TOPCONS [26], and TMHMM (http://www.cbs.dtu.dk/services/TMHMM/), but predicted to have 8 or 9 TMSs using HMMTOP, takes up ribonucleosides and their 2-deoxy derivatives. The topological predictions obtained by the TMHMM program are shown in Figure 3A. It seemed possible that what appears to be TMSs 1–5 and TMSs 6–10 are repeats. It should be noted, however, that topological predictions by the various programs were not consistent, and that some uncertainty exists for this protein and its close homologs. This conclusion did not prevent establishment of the proposed internal repeat.

The RnsC protein was NCBI BLASTed to obtain homologues, which were run through CD-Hit to eliminate redundant and strikingly similar sequences (cut off of 80%). The remaining hits were aligned using the ClustalX program. Using SSearch, putative TMSs 1–5 of all homologues were compared with putative TMSs 6–10. The results showed that homologues in GenBank gi222147212 and gi218884703, probably contain internal five TMS duplications (see Additional file 1: Figure S4A and Figure S4B, respectively). When the first half of gi222147212 was aligned with the second half of gi218884703, a comparison score of 14.9 S.D. with 41.1% similarity and 29.5% identity was obtained (Figure 3B).

#### Internal repeats of 5 TMSs in other 10 TMS transporters, and of 10 TMSs in 20 TMS transporters

**Figure 4:**
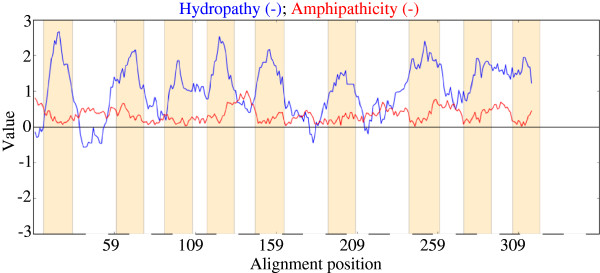
**Hydropathy plot of the BtuC (TC# 3.A.1.13.1) vitamin B**_**12 **_**transport protein.** The topological prediction was performed with the WHAT program. Blue lines denote Hydropathy; Red lines denote Amphipathicity; Orange bars mark transmembrane segments as predicted by HMMTOP.

**Figure 5:**
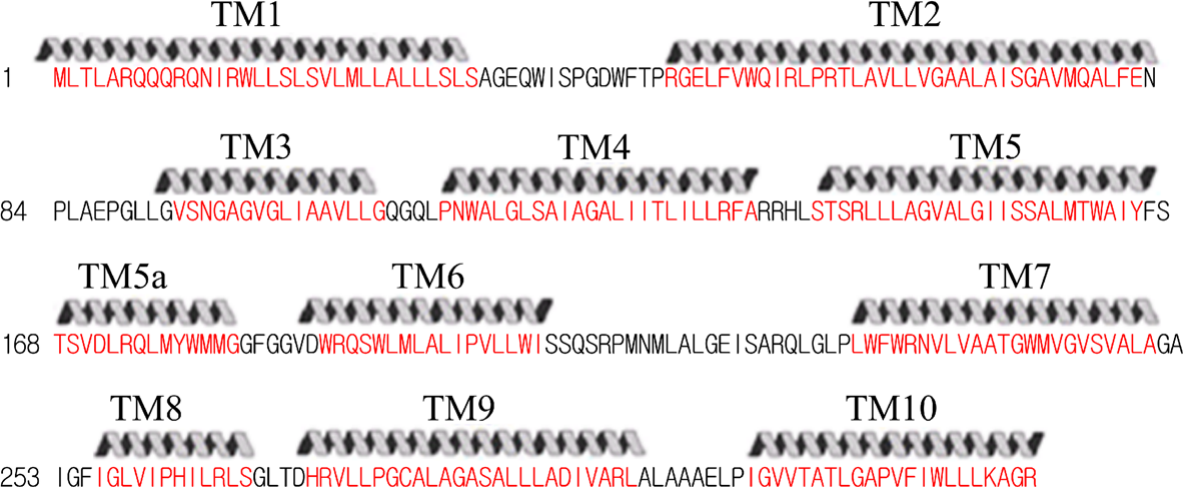
**Red lettering indicates the TMSs (TM1-10) as also indicated by the helical structures above the sequence.** Numbers at the beginning of each line refer to the residue numbers in the protein. TMSs within BtuC revealed by x-ray crystallography.

In this section, we examine other putative 10 TMS proteins and compare predictions with 3-dimensional structures. BtuC (TC# 3.A.1.13.1), a vitamin B_12_ porter constituent, which contains ten TMSs according to the high resolution X-ray crystallographic structure [6], was first examined. However, the WHAT, HMMTOP and TMHMM 2.0 programs all predicted nine TMSs (Figure 4). The topological predictions by WHAT and by X-ray crystallography are shown in Figures 4 and 5, respectively. The missing TMS in Figure 4 is between putative TMSs 7 and 8.

**Figure 6:**
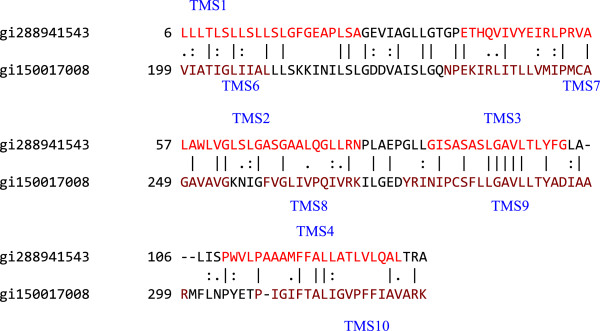
**TMSs 1–4 of gi288941543 aligned with TMSs 6–10 of gi150017008, giving a comparison score of 13.6 S.D. with 42.1% similarity and 31.0% identity.** The numbers at the beginning of each line refer to the residue numbers in each of the proteins. TMSs are indicated in red lettering. Vertical lines indicate identities; colons indicate close similarities, and periods indicate more distant similarities.

The GAP program was run for TMSs 1–4 of gi288941543 aligning with TMSs 6–10 of gi150017008. The result, shown in Figure 6, gave a comparison score of 13.6 S.D. with 42.1% similarity and 31.0% identity. These results clearly show the presence of two internal repeats.

We were able to demonstrate an internal repeat for a twenty TMS transporter, FhuB (TC# 3.A.1.14.3), a protein that catalyzes the transport of iron hydroxamates across the cytoplasmic membrane [27]. Its TMSs 1–10 aligned with TMSs 11–20, as shown in Additional file 1: Figure S5. The comparison score calculated was 33 S.D. with 44.8% similarity and 31.5% identity, demonstrating that TMSs 1–10 and TMS 11–20 resulted from a relatively recent intragenic duplication event.

### Evolutionary relationships among uptake porters with differing numbers of TMSs

In this section, we aim to understand how the ABC uptake porters predicted to contain different numbers of TMSs relate to one another.

#### Understanding the relationships between putative five and six TMS transporters

The five TMS porter investigated in this part of our study is HisM (TC# 3.A.1.3.1), involved in mediating histidine uptake. The hydropathy plot is shown in Additional file 1: Figure S6. A hundred non-redundant homologues of HisM were obtained via BLAST, and the average hydropathy plot, based on the multiple alignment, was derived using the AveHAS program (Additional file 1: Figure S7). The results confirm that HisM is indeed a 5 TMS protein.

To demonstrate the relationship between the five TMS HisM and the six TMS MalG protein, their sequences were aligned. As seen from the alignment shown in Additional file 1: Figure S8, TMSs 2–6 of a MalG homologue, gi239931681, aligned with TMSs 1–5 of a HisM homologue (gi116248748), resulting in a comparison score of 17.5 S.D. (39.2% similarity and 27.9% identity). The extra TMS in MalG, not present in HisM, is therefore TMS1.

TMSs 1–4 of a ten TMS porter, BtuC (TC# 3.A.1.13.1) homologue, gi87122087, aligned with TMSs 1–4 of the six TMS porter, MalG (TC# 3.A.1.1.1) homologue, gi227528545, yielding a comparison score of 11.2 S.D. with 34.9% similarity and 24.8% identity (Additional file 1: Figure S9). These results indicate that in this case, the six TMS porter lost one TMS at its C-terminus to give rise to the five TMS porter. Thus, at least two events gave rise to a 5-TMS topology from a primordial 6 TMS protein, one in which the N-terminal TMS was lost, and one in which the C-terminal was lost.

#### Understanding the relationships between putative six and seven TMS porters

To demonstrate the relationship between transporters that exhibit six or seven predicted TMSs, two proteins were chosen: MalG (TC# 3.A.1.1.1), a six TMS porter, and TogN (TC# 3.A.1.1.11), a putative seven TMS porter. The topological predictions obtained by WHAT and HMMTOP for the latter protein both gave seven TMSs; however, TMHMM predicted this protein to be a six TMS porter. The six TMS topology is also confirmed by TOPCONS and SPOCTUPUS, which according to our unpublished evaluations are the most reliable topological prediction programs currently available. The hydropathy plot of TogN obtained with the WHAT program is shown in Additional file 1: Figure S10.

We obtained the top twenty non-redundant homologues of this protein and used WHAT and TMHMM to predict the topology of each of these homologues. The results are presented in Additional file 1: Table S2. The top twenty non-redundant hits to TogN were examined using the AveHAS program (see TCDB). The average hydropathy plot for these proteins is shown in Additional file 1: Figure S11.

TogN (TC# 3.A.1.1.11), the putative seven TMS porter, aligned with the six TMS MalG homologue, gi134098247. TMSs 1–3 of both proteins aligned, giving a comparison score of 19 S.D. with 30% similarity and 21.9% identity (Additional file 1: Figure S12).

TMSs 4–6 of MalG aligned with TMSs 4–7 of the TogN homologue, gi239820911. The result (Additional file 1: Figure S13) gave a comparison score of 22.4 S.D. with 44.4% similarity and 22.3% identity. We suggest that both proteins have 6 TMSs, and that the 7 TMS prediction is not accurate. Thus, sequences similar to ABC porters predicted to have 7 TMSs may have 6 TMSs.

#### Understanding the relationships between putative six and ten TMS transporters

**Figure 7:**
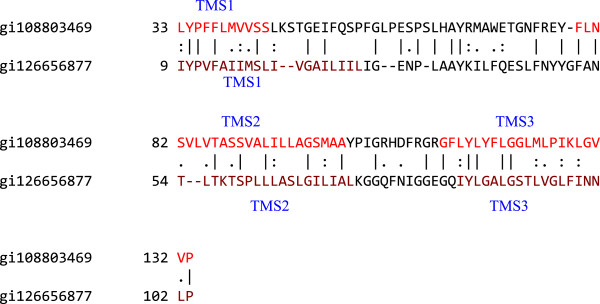
**TMSs 1–3 of gi108803469 aligned with TMSs 1–3 of gi126656877.** The comparison score was 11.2 S.D. with 42.6% similarity and 30.9% identity. The numbers at the beginning of each line refer to the residue numbers in each of the proteins. TMSs are indicated in red lettering. Vertical lines indicate identities; colons indicate close similarities, and periods indicate more distant similarities.

MalG (TC# 3.A.1.1.1), a six TMS transport protein, was aligned with the putative ten TMS protein RnsC (TC# 3.A.1.2.12) to elucidate the relationship between six and ten TMS porters. Homologues of both MalG and RnsC were aligned with MalG and RnsC, respectively, using the GAP and multiple sequence alignment programs to verify that their TMSs aligned in a pattern that would reveal their evolutionary relationships. Then, TMSs 1–3 of a MalG homologue (gi108803469) were aligned with TMSs 1–3 of the RnsC homologue (gi126656877) using GAP. The output gave a comparison score of 11.2 S.D. with 42.6% similarity and 30.9% identity (Figure 7). We conclude that the fourth and fifth TMSs of the RnsC homologue are extra TMSs.

**Figure 8:**
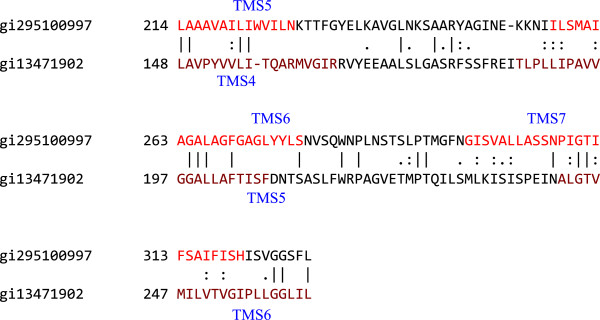
**TMSs 5–7 of gi295100997 aligning with TMSs 4–6 of gi13471902.** The comparison score was 11 S.D. with 32.5% similarity and 20.1% identity. The numbers at the beginning of each line refer to the residue numbers in each of the proteins. TMSs are indicated in red lettering. Vertical lines indicate identities; colons indicate close similarities, and periods indicate more distant similarities.

TMSs 4–6 of a six TMS homologue (gi13471902) aligned with TMSs 6–8 of a putative ten TMS homologue (gi295100997). The result gave a comparison score of 11 S.D. with 32.5% similarity and 20.1% identity (Figure 8). The ninth and tenth TMSs of gi295100997 did not align well with any TMS of gi13471902. Overall, these results indicate that two extra TMSs inserted at the C-terminus of a primordial three TMS protein, followed by an intragenic duplication that gave rise to a ten TMS protein.

**Figure 9:**
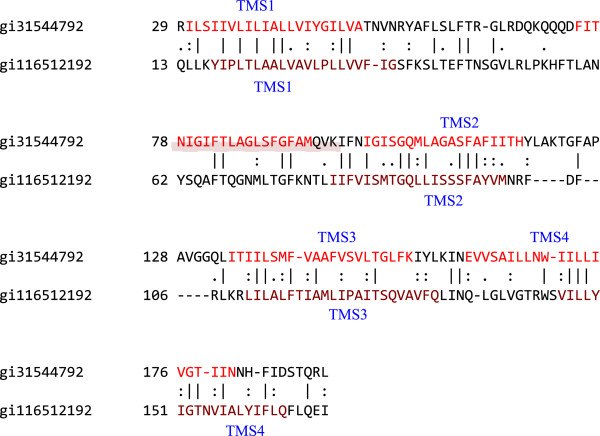
**Putative TMSs 1–4 of an RnsC homologue (gi31544792) (top) aligned with putative TMSs 1–4 of the six TMS MalG homologue (gi116512192) (bottom).** The comparison shown was 12.7 S.D. (45% similarity and 22.5% identity). The numbers at the beginning of each line refer to the residue numbers in each of the proteins. TMSs are indicated in red lettering. Vertical lines indicate identities; colons indicate close similarities, and periods indicate more distant similarities.

In a parallel study, we aligned TMSs 1–4 of the putative 10 TMS RnsC homologue, gi31544792, with TMSs 1–4 of the six TMS MalG homologue, gi116512192. The alignment is shown in Figure 9, resulting in a comparison score of 12.7 S.D. (45% similarity and 22.5% identity). This result suggests that TMS 4 in the 10 TMS protein are from TMS 4 in the 6 TMS precursor before duplication of the 5 TMS unit to give the 10 TMS protein. The proposal that the 5 TMS protein arose by fusion of a 3 TMS unit with a 2 TMS fragment is therefore less probable, for the case of gi31544792. Thus, the last TMS of a 6 TMS homologue may have been lost before duplication to give rise to the 10 TMS homologue. Because of the sequence identity reported in this paragraph, we prefer this last explanation.

#### Understanding the relationships between putative nine and ten TMS transporters

The putative nine TMS protein, HmuU (TC# 3.A.1.14.5), was aligned with the known ten TMS porter, BtuC (TC# 3.A.1.13.1). The sixth TMS from BtuC did not align with a TMS in HmuU. The alignment is shown in Additional file 1: Figure S14. The comparison score is 55.5 S.D. with 52% similarity and 41.4% identity. This high comparison score suggests that the 9 TMS prediction for HmuU is inaccurate; it may have 10 TMSs as does BtuC. It should be recalled that BtuC was also predicted to have 9 TMSs, although the crystal structure revealed 10 TMSs (see above).

#### Understanding the relationships between different ten TMS porters

TMSs 1–5 of a putative ten TMS protein, an RnsC (TC# 3.A.1.2.12) homologue, gi153810044, was aligned with TMSs 1–5 of the ten TMS protein, BtuC (TC# 3.A.1.13.1) homologue, gi73663381, yielding a comparison score of 10.3 S.D. with 32.6% similarity and 22.7% identity (see Additional file 1: Figure S15). Next, TMSs 6–10 of one ten TMS homologue, gi26554040, were aligned with TMSs 1–5 of another ten TMS (TC# 3.A.1.13.1 BtuC) homologue (gi289427840), yielding a comparison score of 10.3 S.D. with 36.4% similarity and 27.9% identity (see Additional file 1: Figure S16). These results show that all five TMSs in the repeat sequences of both proteins can be aligned and exhibit enough similarity to provide evidence of a common origin. It should be noted that inversion of TMSs, hairpin structures and entire protein halves have been documented following alteration of the membrane lipid composition [28], but this appears not to be applicable to the proteins studied here.

#### Understanding the relationships between present-day ABC2 proteins and their ancestral sequence

**Figure 10:**
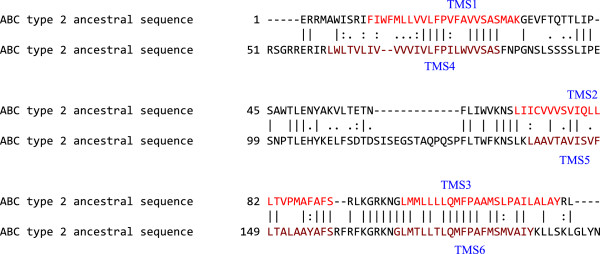
**TMSs 1–3 compared with TMSs 4–6 of an ABC type 2 ancestral sequence.** The comparison score was 39.9 SD with 58.5% similarity and 50.4% identity. The numbers at the beginning of each line refer to the residue numbers in each of the proteins. TMSs are indicated in red lettering. Vertical lines indicate identities; colons indicate close similarities, and periods indicate more distant similarities.

336 homologues of ABC2 uptake systems were extracted from the NCBI protein database using NCBI BLAST. Out of these homologues, those having 6 TMSs were filtered using HMMTOP [29]. 307 of the 336 homologues (top hits) examined were predicted to have 6 TMSs. These proteins were divided into their two halves, each containing three TMSs. Multiple alignments of each unit were achieved using CLUSTALW [30]. Sequences introducing too many gaps in the multiple alignments were removed. ANCESCON was used to construct the root primordial sequence using marginal reconstruction and a maximum likelihood rate factor from alignment-based PI vectors. This program predicts ancestral sequences, usually reliable with confidence levels proportional to the number of homologues available for analysis (unpublished observation). If two proteins, having little sequence similarity derived from a common source, their two ancestral sequences may reveal much greater similarity to each other than any of the present day sequences of the two groups exhibit to each other. Various TMSs within the root primordial sequence (the putative ancestral sequence) as well as the original sequences were subjected to pairwise comparisons using GAP. The comparison scores obtained by GAP are presented in Table 3. Figure 10 shows the GAP comparison of the first half of the ancestral sequence with its second half, resulting in a comparison score of 39.9 standard deviations, 58.4% similarity and 50.5% identity. This confirms the usefulness of the ANCESCON program in predicting ancestral sequences. It also confirms the conclusion that the 3 TMS precursor element duplicated to give rise to the 6 TMS proteins with two 3 TMS repeat units.

**Table 3:** Comparisons between TMSs 1–3 and TMSs 4–6 of ABC2 proteins and their ancestral sequences (see text)

**GI # for TMSs 1-3**	**GI # for TMSs 4-6**	**Present-day ABC2 GAP score (SD)**	**Ancestral sequences GAP score (SD)**
256806846	255254790	11.2	39.9
220933130	255331744	10.5	
256396305	229822375	10.4	

### Structural superposition of MalF and MalG

In Chimera 1.7, we used a function called “MatchMaker” for structural comparisons, always using MalF fragments as reference for all ensuing superimpositions. We iterated by pruning long atom pairs, until no pair exceeded 2 Ångström. For the last 3 TMS superimposition, the result was excellent. We saved the superimposed structures in a single file. In the “Reply Log”, we could see that the RMSD between 54 atom pairs was 1.156 Ångström. There is a slight shift, based on the start point of the superimposition, giving slightly higher RMSD values for the last 2 TMSs.

The motif “DxW+LAL” is located at the beginning of the long insert in MalF and also in a short insert between TMS1 and TMS2 in MalG. The presence of the short insert between TMS1 and TMS2 in MalG, and the presence and location of this motif, would suggest that it is the first two TMSs in MalF that should be “chopped” or considered as the “extra” TMS pair. The superimposition between TMS 3, 4 and 5 in MalF that corresponds to TMS 1, 2 and 3 in MalG resulted in an RMSD between 37 atom pairs of 0.880 Ångström, confirming our assumptions.

To facilitate sequence comparison between the first domain duplicated 3 TMS unit in MalF and MalG, we removed parts of the long insert in MalF (RYV … LSA), and based on the presence of 17 residues after the DxW+LAL motif in MalG, we removed 124 amino acyl residues (GEQ … IQK). We also took out the sequence (MAM … GEY). After this editing, the respective sequences had the lengths 166 and 151. Using Protocols 1 and 2, we found that this comparison resulted in a GSAT Z-score of 21 S.D. The importance of the DxW+LAL motif was that it was the only motif conserved between the two sequences that we discovered when we compared MalF and MalG. It was important because it helped to establish correspondance between the long insert in MalF and a shorter, but still extended, loop in MalG.

**Figure 11:**
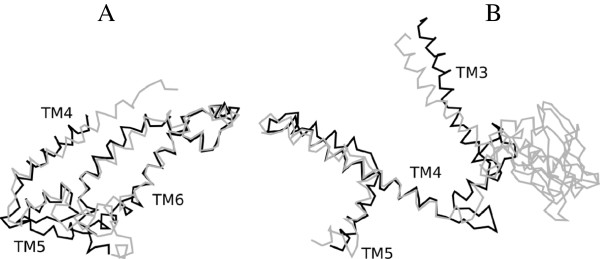
**Structural superimposition of MalF and MalG. A** (left). The last 3 TMS domain-duplicated unit of MalF (TMSs 6, 7 and 8) superposed on that of MalG (TMSs 4, 5 and 6). The TMS numbering shown is taken from MalG. The light colored chain represents MalG, and the coordinates used are the X/Y coordinate columns. **B** (right). The first 3 TMS domain-duplicated unit of MalF (TMSs 3, 4 and 5) superposed on the first duplicated unit of MalG (TMSs 1, 2 and 3). The TMS numbering shown is for MalF. The light colored chain represents MalF, and the coordinates used are the Y/Z coordinate columns.

In Chimera, we attempted a superposition of the first and last 3 TMSs of MalG, using the last 3 TMSs as the reference for superimposition (Figure 11). For MalF, we took the last 3 TMSs, and then 270–350 only (this is domain unit 1, only 2 TMSs after the insert). We repeated this, but without removing the insert, using residues 65–350 as the reference. These tests failed and did not result in a credible superimposition.

The start and end of MalF generated two lists from Protocol 1 each. Analyzing these lists in Protocol 2 revealed that they contain many identical hits, the highest scoring common entry being “Sba1”, scoring 396 against itself in GSAT. This may be the expected outcome when we analyze parts of the same sequence. To better evaluate similarity between the first and second 3 TMS units, we took the first half from MalG and the final 3 TMSs from MalF. For this comparison, we observed a comparison score of 21 S.D.

To compare our interpretation that MalF has 2 additional TMSs at its N-terminus, a long insert between TMSs 3 and 4, and that it differs from the other proteins that have a putative 10 TMS structure (5 + 5 TMS), such as RnsC which is discussed at length in this report, we used Protocol1 to generate a list of RnsC homologues. We then used Protocol2 to compare MalF and RnsC. In fact, the best scoring pair between RnsC and MalF scored 12 S.D., but careful examination of the GSAT alignment showed that the TMSs did not align well. While 8 sequence pairs scored 10 S.D. or greater, the actual alignments did not cover the full sequence length and contained misaligned TMS segments. This illustrates the point that these sequences are not closely related in spite of their distant sequence similarities that presumably reflect their common origin. Furthermore, while we consider RnsC to be a 5 + 5 TMS protein, some programs such as TMHMM predict 8 or 9 TMSs, having 2 weak TMS predictions between TMS 2 and 3 in both of the domain duplicated units. This uncertainty has been discussed in detail above.

#### Possible origin of ABC1 porters from ABC2 porters

**Figure 12:**
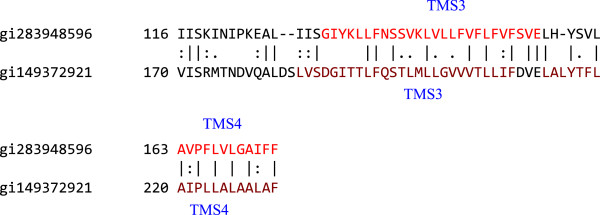
**Possible origin of ABC1 porters from ABC2 porters.** TMSs 3 and 4 of an ABC1 homologue, gi283948596 (top), aligned with TMSs 3 and 4 of an ABC2 homologue, gi149372921 (bottom), giving a comparison score of 11 S.D, 52.5% similarity and 39% identity. The numbers at the beginning of each line refer to the residue numbers in each of the proteins. TMSs are indicated in red lettering. Vertical lines indicate identities; colons indicate close similarities, and periods indicate more distant similarities.

Many ABC1 porters were aligned with many ABC2 porters. In almost all cases (~80%), TMSs 3 and 4 in the ABC1 porters aligned with TMSs 3 and 4 in the ABC2 porters as the high scoring pairwise comparisons. The alignment of TMSs 3 and 4 from the type I porter protein, gi283948596, and the type II porter protein, gi149372921, is shown in Figure 12. This alignment resulted in a comparison score of 11 S.D. with 52.5% similarity and 39% identity. The results indicate that ABC1 and ABC2 proteins are somehow related, although the possibility of convergent sequence similarity must be considered as an alternative explanation, given the short lengths of the sequences being compared.

The fact that the TMSs shared are 3 and 4 in both proteins, where 3–4 of ABC2 are the last and first TMSs of the two repeat sequences, while TMSs 3–4 of ABC1 comprise the central 2 TMS repeat unit, suggested that if these TMSs do exhibit this degree of sequence similarity due to divergent evolution from a common ancestral sequence, ABC2 proteins must have preceded ABC1 proteins. However, the shortness of the sequences compared (50 amino acids) renders this conclusion tentative. Regardless, from x-ray crystallographic studies, it is clear that ABC1 and ABC2 proteins do not have a common fold, and therefore have not retained 3-dimensional structural features as expected [6,7].

To understand why TMSs 3 and 4 of both transporter types proved to show the greatest sequence similarity, the three repeat units in ABC1 porter were examined. The results revealed that sequence divergence of the first and third repeats was greater than that of the central repeat (Table 4). This observation could explain why the central repeats of ABC1 porters were recognized as similar to the potential precursors, TMSs 3 and 4 of ABC2 porters, while the first and third repeats were not.

**Table 4:** Comparisons between TMSs 3 and 4 of Type 1 (ABC1) and Type 2 (ABC2) proteins

**TC # (ABC2)**	**TC # (ABC1)**	**GAP score in standard deviations**
3.A.1.101.1	3.A.1.109.1	12
3.A.1.101.1	3.A.1.212.1	10.6
3.A.1.101.1	3.A.1.206.1	12.5
3.A.1.101.1	3.A.1.113.1	10.8
3.A.1.101.1	3.A.1.208.1	12.6
3.A.1.127.1	3.A.1.106.1	11.1
3.A.1.102.1	3.A.1.106.1	12.1

## Discussion

### Essentially all ABC uptake transporters are homologous

**Figure 13:**
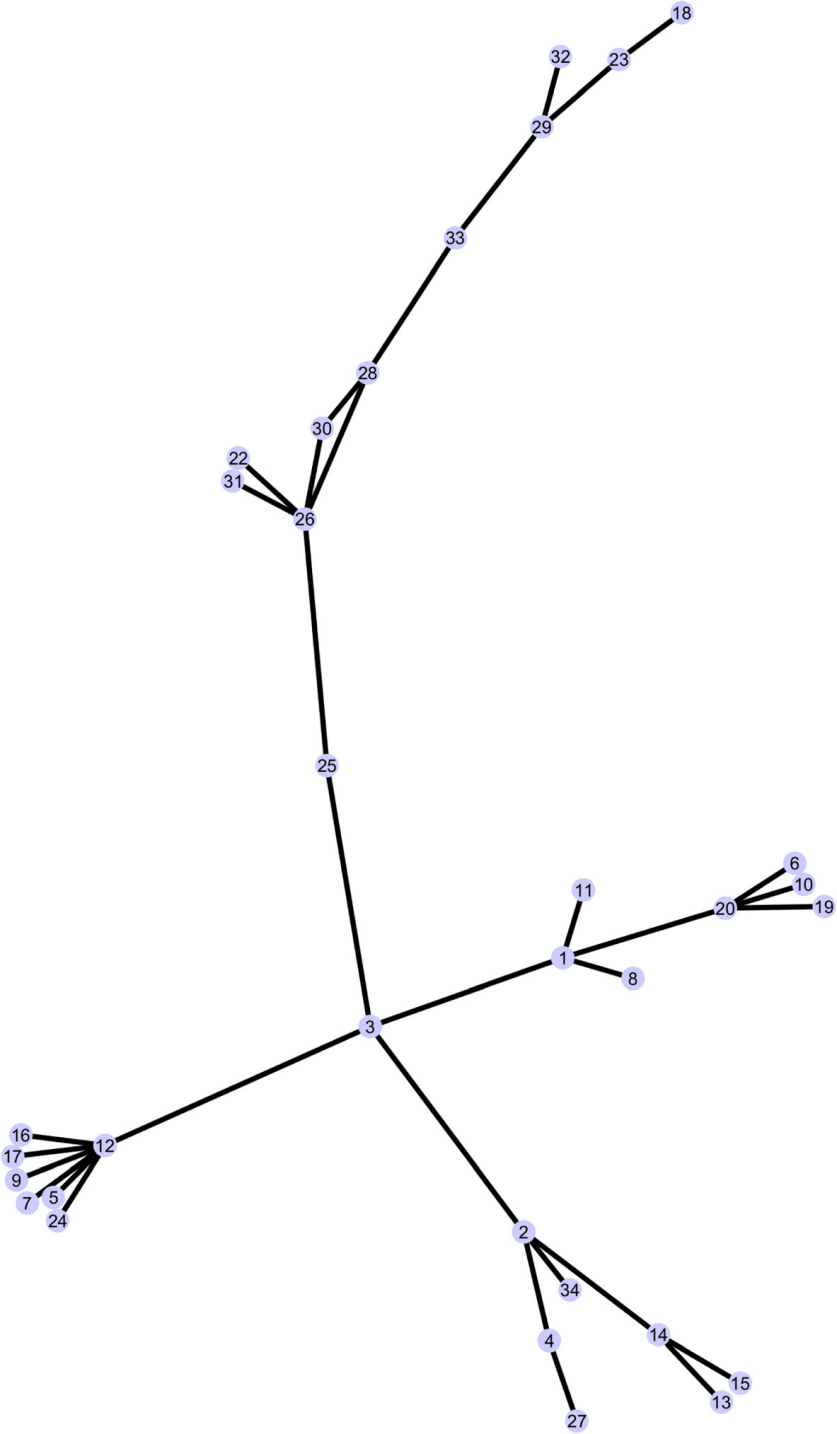
Cytoscape 2.8.3 graph, using spring embedded logic, of significant relationships between all families within 3.A.1.

The results reported in Table 1 (and visualized in Figure 13) provide statistical evidence that all 35 families of ABC uptake porters, except family 21, contain integral membrane proteins that are homologous to each other. They are believed to have arisen from a 3 TMS precursor which duplicated to give 6 TMS porters, many of which are represented in present day integral membrane uptake and export transport systems. However, although alternative topological variants have arisen (5, 10, 12 and 20 TMSs, and possibly 7, 8 and 9 TMSs as well), we could demonstrate homology using a cut-off point of 10 (or more) S.D. for a stretch of at least 60 continuous amino acyl residues. Because of the tremendous topological variation, we do not expect all of these proteins to exhibit the same 3-dimensional folds although so far, this has been the case. The one exceptional family of solute uptake porters that could not be shown to be homologous to other ABC uptake porters was family 21, which clearly is related to ABC1 export proteins.

### Topological uncertainties

The ABC uptake transporters whose X-ray structures were available at the time of writing are the vitamin B_12_ porter of *E. coli* (BtuCDF, TC# 3.A.1.13.5) [6], the probable metal chelate uptake system of *Haemophilus influenzae* (HI1471, TC# 3.A.1.14.11) [31], the methionine transporter of *E. coli* (MetNI, TC 3.A.1.24.1) [7], the maltose porter of *E. coli* (MalEFGK, TC# 3.A.1.1.1) [32] and the molybdate porter of *Methanosarcina acetivorans* (ModABC, TC# 3.A.1.8.2) [33]. All of these transport systems have similar folds in agreement with our understanding that these uptake systems (except family 21) derived from a common ancestor. This fold differs from that of the ABC1 efflux porters for which x-ray structures are available [1].

The topological predictions obtained by the WHAT and TMHMM programs indicated that MalG (TC# 3.A.1.1.1) is a six TMS porter, in agreement with the X-ray structural data [7]. However, the vitamin porter, BtuC (TC# 3.A.1.13.1), and HI1471 were both predicted to contain 9 TMSs by both programs, and TOPCONS, yet the X-ray structures shows there to be 10 [6]. Both ModB and MetI were predicted to have 5 TMSs using all three programs, and the X-ray structures confirmed this conclusion. No such data are available for the histidine permease protein, HisM from *Salmonella typhimurium*. The topologies predicted by WHAT, TOPCONS and TMHMM for this porter are 5, 5 and 4 TMSs, respectively. Similar disagreements occurred for several other uptake porters (Additional file 1: Table S3). Overall, our data suggest that the topological predictions obtained using the standard bioinformatic programs are helpful but not fully reliable. Average hydropathy plots, obtained using the AveHAS program for members of a family should be used for more reliable topological predictions when conflicting topological predictions arise. This practice was followed here. While some families of transporters give consistently reliable predictions with programs such as HMMTOP and TMHMM (e.g., MFS (TC# 2.A.1) and APC (TC# 2.A.3) family members), some such as members of the largely eukaryotic Mitochondrial Carrier Family (2.A.29), the ubiquitous Trk family and the prokaryotic-specific phosphoenol-pyruvate sugar phosphotransferase system (PTS; TC# 4.A) do not [34].

Since almost all ABC uptake systems proved to be homologous to ABC2 efflux systems, it is possible that ABC2 efflux systems were the precursors of these uptake systems. However, evidence for this postulate is weak. The argument depends in part on the fact that efflux systems are ubiquitous while uptake systems are essentially lacking in eukaryotes. An alternative postulate will be presented elsewhere (EI Sun and MH Saier, manuscript in press). We propose that ABC2 porters were primordial proteins that predated both ABC1 and ABC3 proteins, but this postulate is highly speculative.

### Proposed pathway for the appearance of ABC uptake systems

**Figure 14:**
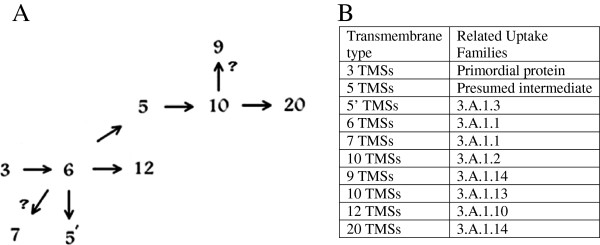
**Proposed evolutionary pathway and primordial sequences of the different topological types of ABC uptake systems. A** (left). The proposed evolutionary pathway for the appearance of present-day ABC uptake systems. **B** (right). Presumed primordial or intermediate sequences and representative examples of the different topological types of ABC transmembrane porter proteins.

Our proposed pathway for the appearance of ABC uptake systems of differing topologies is shown in Figure 14. A primordial 3 TMS porter duplicated internally to give rise to a 6 TMS porter [1], and this 6 TMS porter again duplicated to give rise to a 12 TMS porter. Possibly a primordial 4 TMS porters could have arisen via either of two routes: first, one TMS might have been added at the C-terminus of the three TMS precursor, or second, the six TMS porter could have lost two TMSs at its C-terminus. Although speculation in view of the uncertainties of the topological predictions, the second route is favored (see Results). Further, one TMS could have been added between the 5th and 6th TMSs of a 6 TMS porter to give rise to a 7 TMS porter; however, the occurrence of this 7 TMS topological type is less likely and may be due to erroneous predictions by the HMMTOP and TMHMM programs.

Starting with similar 3 TMS internally duplicated primordial 6 TMS porters, one TMS was apparently deleted at the N-terminus to gives rise to some of the current 5 TMS porters. In a distinct event, a 6 TMS porter may have lost a C-terminal TMS to give rise to a different 5 TMS type of porter. These two events, giving rise to two recognizably distinct 5 TMS homologues, undoubtedly occurred independently of each other as indicated in Figure 14.

Although likely, it is not absolutely certain that a 6 TMS protein gave rise to the C-terminally truncated 5 TMS homologue in a single step. Possibly, the 5 TMS protein arose in two steps via a 4 TMS intermediate. Four-TMS ABC uptake porter proteins could have existed [12] as their 8 TMS duplicated products may exist today, but this suggestion is not well documented. Because TMSs 5 in the 5 TMS homologues do not show appreciable sequence similarity with TMS 5 in the 6 TMS proteins (Figure 10), we cannot securely distinguish the route from a 6 TMS or a 4 TMS precursor. However, the simpler one step pathway is favored. Intragenic duplication of a 5 TMS homologue gave rise to the 10 TMS porters, and the 10 TMS porter duplicated internally to give rise to the 20 TMS porters. Aligning the first ten TMSs with the second ten TMSs of the twenty TMS porters yielded high comparison scores (≥ 33 S.D.), indicating that this intragenic duplication event happened relatively recently in evolutionary time.

We have succeeded in using public structural data to show that TMSs 1 and 2 are the two “extra” helices in the MalF sequence compared to MalG. This means that MalF differs from MalG in two overarching ways, by having the two additional TMSs at the start of the sequence, and secondly, by having a much longer insert between TMS 3 and 4. However, we also noted that the MalG sequence may contain a small insert in the corresponding position between TMSs 1 and 2.

We have used Protocol2 to confirm that, for the last three TMSs, there is equivalence between MalF and MalG. The GSAT Z-score was 21 S.D. for the best scoring pair of related sequences found using Protocol1. This is far in excess of what is required to establish homology. Comparisons between MalF and MalG, using programs such as ClustalW2 is complicated because of the long insert. Pairwise BLASTP searches identified a couple of motifs, such as “DxW+LAL”, but the sequence similarity was not obvious outside of these motif regions. This can perhaps be compared with cases of homology modeling of orthologous proteins between closely related species, where structure modeling is attempted based on highly similar sequences and result in comparable RMSD scores of <1 for sequences of length ~100.

The partial sequences for MalF and MalG have very similar folds, apparent in the superpositions presented here, where the domain-duplicated 3 TMS units resulted in RMSD values near or below 1. The general value of this comparison is illustrated by establishment of a reference point for interpretation of GSAT scores using structural comparisons. Thus, we have shown that very similar folds correspond to sequence similarity resulted in GSAT scores above twenty. It is clear that the modifications (insertions/fusion) that gave rise to the 8 TMS MalF from a 6 TMS MalG-like precursor occurred after the duplication of 3 TMSs to give 6 TMSs, but the duplication of the 5 TMS precursor to give 10 TMS proteins occurred after the loss of an N- or C-terminal TMS from the 6 TMS precursor.

## Conclusion

In summary, the results reported in this communication are consistent with our more general conclusion that most ABC uptake integral membrane proteins arose from the basic ABC2 topology modified by a variety of insertions/deletions (indels) which sometimes occurred before duplication generating the full-length proteins as documented in several examples. Sometimes these occurred after this duplication event occurred, as documented for MalF. It seems clear that during the evolution of ABC uptake proteins, these intragenic duplication events occurred multiple times as also suggested for other families of transporters [16].

## Methods

### Statistical analyses

The binary comparisons presented in the Results section were the ones that of those examined gave the largest comparison scores. The TMSs compared were in general determined from the hydropathy plots, but in those cases where 3D structures were available, they were determined from the 3D structures.

The query sequences of the integral membrane proteins of uptake transporters were retrieved from the Transporter Classification Database (http://www.tcdb.org). To establish homology (common ancestry), either between two proteins or between two internal segments in a set of homologous proteins, the SSearch, IC and GAP programs were initially used [13,14,21,35]. To establish homology among putative full-length homologues or repeat sequences of greater than 60 amino acyl residues, a value of 10 standard deviations (S.D.) was considered sufficient [4,18]. According to Dayhoff *et al.*[36], this value corresponds to a probability of 10^-24^ that this degree of similarity arose by chance [36].

We have found that a single iteration with a cut-off value of e^-4^ for the initial BLAST search, and a cut-off value of e^-5^ for the second iteration, reliably retrieves homologues with few false positives. Nevertheless, all proteins giving BLAST e-values of e^-7^ or larger were tested for homology using the GAP program with default settings, requiring a comparison score of at least 10 S.D. in order to conclude that these proteins share a common origin. All hits that satisfied these criteria were put through a modified CD-Hit program with a 90% cut-off value [13,24] to eliminate redundancies, fragmentary sequences and sequences with greater that 90% identity with a kept protein.

gi-Extract from TCDB was used to extract the gi numbers of homologues, which were then searched through NCBI to obtain the FASTA sequences. A multiple alignment was generated with the ClustalW2 program, and homology of all aligned sequences throughout the relevant transmembrane domains was established using the SSearch and GAP programs [13,21,35]. Internal regions were examined for repeats whose dissimilar segments were compared with potentially homologous regions of the same proteins using the SSearch and GAP programs with default settings. The ATP hydrolyzing (ABC) domains of these systems were excluded, and only the transmembrane domains or proteins were used in the analyses.

### Topological analyses

Average hydropathy, amphipathicity and similarity plots for multiply aligned sets of homologues were generated with the AveHAS program [37], while web-based hydropathy, amphipathicity and predicted topology for an individual protein were estimated using the WHAT program [25] as well as the TMHMM 2.0 [38], HMMTOP [29], and TOPCONS [topcons.cbr.su.se/] programs. Some of these programs were updated as described by Yen *et al.*[13,21]. Sequences were spliced for statistical analyses as described by Zhou *et al.*[15]. The global alignment program with displayed TMSs (GAP-DT), in combination with the SSearch and GAP programs, was used to determine where an extra transmembrane domain might have been inserted into or added to a transporter of a smaller number of TMSs to give rise to a transporter with a larger number of TMSs. ANCESCON, which takes a multiple sequence alignment as the input [39], is an ancestral sequence reconstruction program used to construct putative ancestral sequences of a group of homologous proteins. The root primordial sequence was constructed using the marginal reconstruction algorithm.

### Superimpostion using Chimera

We loaded chains F and G (MalF and MalG of the maltose transporter from *E. coli* K12) from PDB (# 2R6G) into UCSF Chimera 1.7 (http://www.cgl.ucsf.edu/chimera/). Initial TMS predictions were taken from TMHMM 2.0 (http://www.cbs.dtu.dk/services/TMHMM/), and compared with the Protein Feature View at (http://www.rcsb.org/pdb/explore/explore.do?structureId=2R6G) for the F and G chains. The following approximate positions of the TMSs were used. MalF: 20–40; 40–60; 70–90; gap; 280–300; 320–340; 370–390; 430–450; 490–510. MalG: 20–40; 90–110; 120–140; middle; 155–175; 210–230; 260–280. The actual PDB file was downloaded and edited, so that it only contained the lines starting with “ATOM”. We cut out the last 3 TMSs from each chain (MalF 360–504 and MalG 145–290) and transferred these to a new location.

### Motif identification

To search for matching segments between MalF and MalG, we blasted the sequence pair against each other and identified a motif, “EA + A + DGA”, located between TMS 1 and TMS 2 in the last 3 TMS segments of both MalF and MalG. We also identified other motifs, including “FPL+”, “+AI”, “SW”, and “DxW+LAL”. To confirm the hypothesis that it is TMSs 3, 4 and 5 in MalF that correspond to TMSs 1, 2 and 3 in MalG, we extracted the following atom coordinate sets from the "2R6G" model: 65 – 350 in MalF and 10 – 150 in MalG. These alpha carbon traces were superposed in Chimera in the same way as previously described.

### Ancient Rep

To compare our results using Protocol 1 and Protocol 2, we focused on the last 3 TMSs in MalF and MalG. These sequences have a common fold, but the sequence similarity is not apparent. We took sequences from LFG … KFD in MalF, and sequence from IPF … to VKG in MalG. These were entered into Protocol 1 [16], setting CD-HIT to 0.8. In Protocol 2, the best scoring pair for the comparison of two lists of hits from an iterative search based on the last 3 TMSs in MalF and MalG, had a GSAT Z-score of 21 S.D., far in excess of what is required to establish homology. Protocols 1 and 2 are standard tools, part of the BioV Suite, reported by Reddy and Saier (2012). Protocol 1 runs a PSI-BLAST search with iterations, collects results, removes redundant/similar sequences, annotates, tabulates, and counts TMSs. Protocol 2 allows the rapid identification of homologs between any two FASTA files using the G-SAT program also described by Reddy and Saier [16].

To elucidate the domain duplication history of MalG, we ran Protocol 1 on MalG in preparation for running ANCIENT REP [16]. We took P68183 from http://www.tcdb.org/search/result.php?tc=3.A.1.1.1, not counting TMSs, using “test” as the output path, and 0.8 as the CD-HIT threshold. We then used “ancient -i results.faa -r 3 -o test2 –method = 3 –threads = 4”. We repeated for MalF. The following topology categories were created '1-2-3 2-3-4 3-4-5 4-5-6 5-6-7'. The '5-6-7' topology category was created because while MalG has a 3 + 3 TMS structure, it is related to some putative 7 TMS sequences. For MalG, none of the sequences in ‘horizontal.txt’ produced a high GSAT Z-score [16]. The three best hits were: Tra1 (4 S.D.), Opr1 (4 S.D.), and Dra1 (5 S.D.).

None of the results for the horizontal method scored high, the highest was only 5 S.D. (for 3-4-5 and 6-7-8 in Tfu1). The following topology categories were created '1-2-3 2-3-4 3-4-5 4-5-6 5-6-7'. There were 1084 results that scored 10 or better in the '1-2-3' topology category. In the '2-3-4' topology category, 1061 proteins scored 10 or better, and in the '3-4-5' topology category, 994 sequence pairs scored 10 or better. There were 615 protein pairs that scored better than 10 in the '4-5-6' topology category. In the '5-6-7' topology category, only 101 protein pairs scored better than 10, pairing with TMS 8-9-10 of the other proteins. According to our previous results, MalF should score highest against a model where TMS 3-4-5 matches TMS 6-7-8. This is in agreement with the sharp drop in sequence pairs in the 5-6-7 topology category and supports our conclusions.

## Authors’ contributions

Conceived and designed the experiments MHS; Performed the experiments WHZ, MAS, EIS; Analyzed the data: WHZ, AV, MHS; Contributed reagents/materials/analysis tools VR; Wrote the paper MHS, WHZ, MAS, EIS. All authors read and approved the final manuscript.

## Supplementary Material

Additional file 1 Supplementary Tables and Figures. Click here for file
