# Supplementary material for: Evolutionary relationships of ATP-Binding Cassette (ABC) uptake porters
Source: BMC Microbiol. 2013 May 6;13:98. doi: 10.1186/1471-2180-13-98 (PMC3654945; doi:10.1186/1471-2180-13-98)
Supplement: Additional file 1 — Supplementary Tables and Figures. [file 1471-2180-13-98-S1.docx]

**Supplementary Tables S1-S3**

Table S1. Partial TC Blast output of 3.A.1.1.1 MalF

| Sequences producing significant alignments: | Score | E-Value |
| --- | --- | --- |
| [P02916](http://www.tcdb.org/search/result.php?acc=P02916&tc=3.A.1.1.1) 8 TMSs [3.A.1.1.1](http://www.tcdb.org/search/result.php?tc=3.A.1) Maltose transport system permeas... | [946](http://www.tcdb.org/progs/blast.php#P02916) | 0.0 |
| [Q9X0T0](http://www.tcdb.org/search/result.php?acc=Q9X0T0&tc=3.A.1.1.22) 8 TMSs [3.A.1.1.22](http://www.tcdb.org/search/result.php?tc=3.A.1) Maltose ABC transporter, permea... | [256](http://www.tcdb.org/progs/blast.php#Q9X0T0) | 7e-69 |
| [O32261](http://www.tcdb.org/search/result.php?acc=O32261&tc=3.A.1.1.2) 9 TMSs [3.A.1.1.2](http://www.tcdb.org/search/result.php?tc=3.A.1) Putative arabinogalactan oligome... | 176 | 5e-45 |
| [O06990](http://www.tcdb.org/search/result.php?acc=O06990&tc=3.A.1.1.26) 8 TMSs [3.A.1.1.26](http://www.tcdb.org/search/result.php?tc=3.A.1) Putative uncharacterized protei... | [165](http://www.tcdb.org/progs/blast.php#O06990) | 2e-41 |
| [Q48396](http://www.tcdb.org/search/result.php?acc=Q48396&tc=3.A.1.1.6) 8 TMSs [3.A.1.1.6](http://www.tcdb.org/search/result.php?tc=3.A.1) CYM A,B,C,D,E,F,G,H,I,J GENES - ... | [146](http://www.tcdb.org/progs/blast.php#Q48396) | 6e-36 |
| [Q8DT27](http://www.tcdb.org/search/result.php?acc=Q8DT27&tc=3.A.1.1.27) 8 TMSs [3.A.1.1.27](http://www.tcdb.org/search/result.php?tc=3.A.1) Putative maltose/maltodextrin A... | [118](http://www.tcdb.org/progs/blast.php#Q8DT27) | 2e-27 |
| [Q8TZP9](http://www.tcdb.org/search/result.php?acc=Q8TZP9&tc=3.A.1.1.16) 6 TMSs [3.A.1.1.16](http://www.tcdb.org/search/result.php?tc=3.A.1) Putative sugar transport inner ... | [112](http://www.tcdb.org/progs/blast.php#Q8TZP9) | 1e-25 |
| [O51924](http://www.tcdb.org/search/result.php?acc=O51924&tc=3.A.1.1.7) 8 TMSs [3.A.1.1.7](http://www.tcdb.org/search/result.php?tc=3.A.1) INNER MEMBRANE PROTEIN MALF - Th... | [106](http://www.tcdb.org/progs/blast.php#O51924) | 9e-24 |
| [Q8KN18](http://www.tcdb.org/search/result.php?acc=Q8KN18&tc=3.A.1.1.33) 6 TMSs [3.A.1.1.33](http://www.tcdb.org/search/result.php?tc=3.A.1) Putative sugar transporter inte... | [89](http://www.tcdb.org/progs/blast.php#Q8KN18) | 2e-18 |
| [Q9R9Q6](http://www.tcdb.org/search/result.php?acc=Q9R9Q6&tc=3.A.1.1.17) 8 TMSs [3.A.1.1.17](http://www.tcdb.org/search/result.php?tc=3.A.1) ThuF (Probable trehalosemaltose... | 87 | 6e-18 |
| [P10905](http://www.tcdb.org/search/result.php?acc=P10905&tc=3.A.1.1.3) 6 TMSs [3.A.1.1.3](http://www.tcdb.org/search/result.php?tc=3.A.1) SN-glycerol-3-phosphate transpor... | 86 | 2e-17 |
| [Q8L125](http://www.tcdb.org/search/result.php?acc=Q8L125&tc=3.A.1.1.19) 6 TMSs [3.A.1.1.19](http://www.tcdb.org/search/result.php?tc=3.A.1) PalF - Agrobacterium tumefaciens. | [85](http://www.tcdb.org/progs/blast.php#Q8L125) | 2e-17 |
| [Q97ZC2](http://www.tcdb.org/search/result.php?acc=Q97ZC2&tc=3.A.1.1.15) 6 TMSs [3.A.1.1.15](http://www.tcdb.org/search/result.php?tc=3.A.1) Maltose transport inner membran... | 84 | 6e-17 |
| [Q72H67](http://www.tcdb.org/search/result.php?acc=Q72H67&tc=3.A.1.1.25) 6 TMSs [3.A.1.1.25](http://www.tcdb.org/search/result.php?tc=3.A.1) Maltose transport system permea... | 83 | 1e-16 |
| [O30832](http://www.tcdb.org/search/result.php?acc=O30832&tc=3.A.1.1.5) 6 TMSs [3.A.1.1.5](http://www.tcdb.org/search/result.php?tc=3.A.1) SORBITOL/MANNITOL TRANSPORT INNE... | 79 | 2e-15 |
| [Q8RJU9](http://www.tcdb.org/search/result.php?acc=Q8RJU9&tc=3.A.1.1.18) 6 TMSs [3.A.1.1.18](http://www.tcdb.org/search/result.php?tc=3.A.1) Transmembrane protein - Strepto... | 77 | 6e-15 |

Table S2. Topological predictions of homologues of the putative 7 TMS uptake porter, TogN, obtained by the WHAT and TMHMM programs.

| Homologue gi | WHAT | TMHMM |
| --- | --- | --- |
| 307131160 | 7 TMSs | 6 TMSs |
| 283786544 | 7 TMSs | 6 TMSs |
| 227327812 | 7 TMSs | 6 TMSs |
| 152971776 | 7 TMSs | 6 TMSs |
| 260599192 | 7 TMSs | 6 TMSs |
| 22125776 | 7 TMSs | 6 TMSs |
| 88800131 | 7 TMSs | 6 TMSs |
| 260769972 | 7 TMSs | 6 TMSs |
| 152994809 | 7 TMSs | 6 TMSs |
| 150388730 | 6 TMSs | 6 TMSs |
| 241772502 | 6 TMSs | 6 TMSs |
| 237736869 | 6 TMSs | 6 TMSs |
| 269122634 | 6 TMSs | 6 TMSs |
| 260219973 | 6 TMSs | 6 TMSs |
| 269119325 | 6 TMSs | 6 TMSs |
| 289578916 | 6 TMSs | 6 TMSs |
| 114761276 | 7 TMSs | 6 TMSs |
| 15891797 | 7 TMSs | 6 TMSs |
| 296442667 | 6 TMSs | 6 TMSs |
| 304317803 | 7 TMSs | 6 TMSs |
| 266623373 | 6 TMSs | 6 TMSs |
| 257874830 | 6 TMSs | 6 TMSs |

Table S3. Comparison of topological predictions for transmembrane proteins obtained with the WHAT and TMHMM programs.

| TC# | WHAT  prediction | TMHMM  prediction |
| --- | --- | --- |
| HisM (3.A.1.3.1) | 5TMSs | 4TMSs |
| MalG (3.A.1.1.1)  (established to have 6TMSs) | 6TMSs | 6TMSs |
| TogN (3.A.1.1.11) | 7TMSs | 6TMSs |
| RnsC (3.A.1.2.12) | 8TMSs | 9TMSs |
| HmuU (3.A.1.14.5) | 9TMSs | 8TMSs |
| BtuC (3.A.1.13.1)  (established to have 10TMSs) | 9TMSs | 9TMSs |
| FutB (3.A.1.10.2) | 12TMSs | 12TMSs |
| FhuB (3.A.1.14.3) | 20TMSs | 17TMSs |


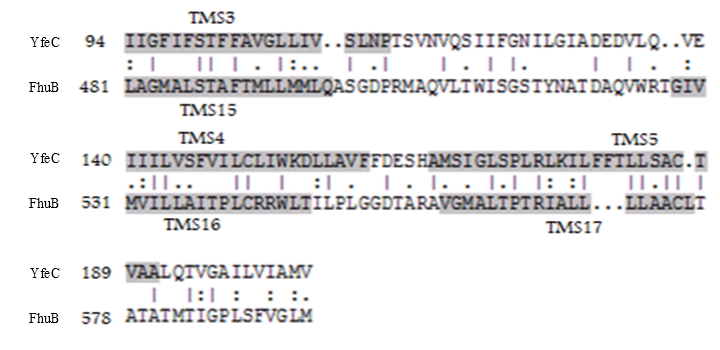


Fig. S1. TMS alignment between YfeC (3.A.1.15.4) and FhuB (3.A.1.14.3). The comparison score was 18 S.D. with 39.8% similarity and 29.6% identity.


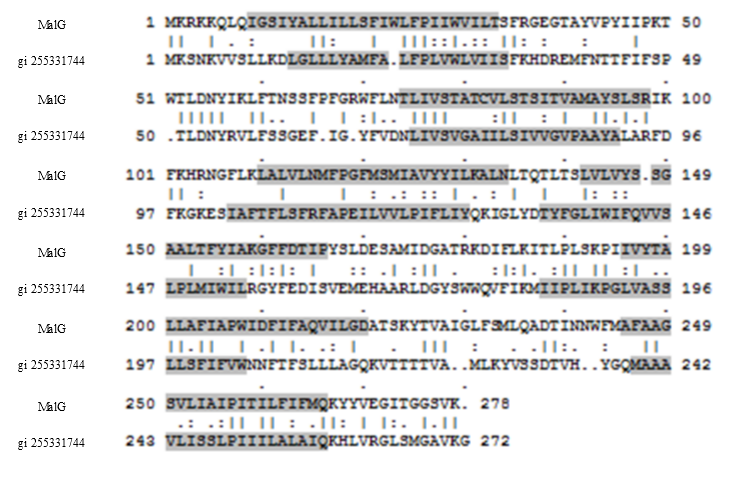


Fig. S2A. Alignment of MalG with gi 255331744 yielding a comparison score of 43 S.D. with 46% similarity and 31% identity.


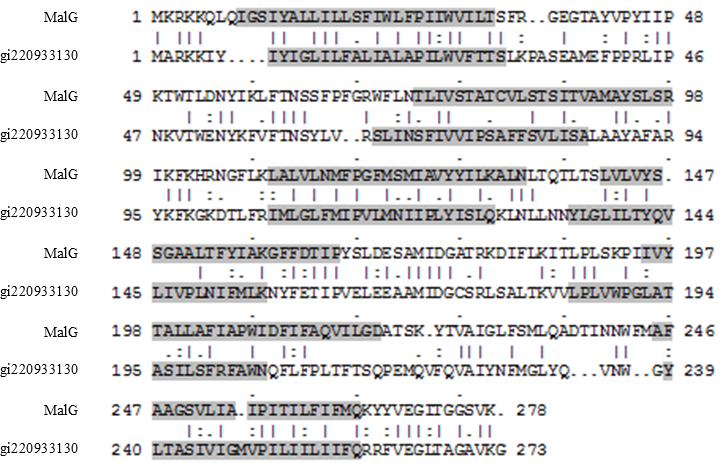


Fig. S2B. Alignment of MalG with gi220933130 yielding a comparison score of 48 S.D. with 46.8% similarity and 37.1% identity.


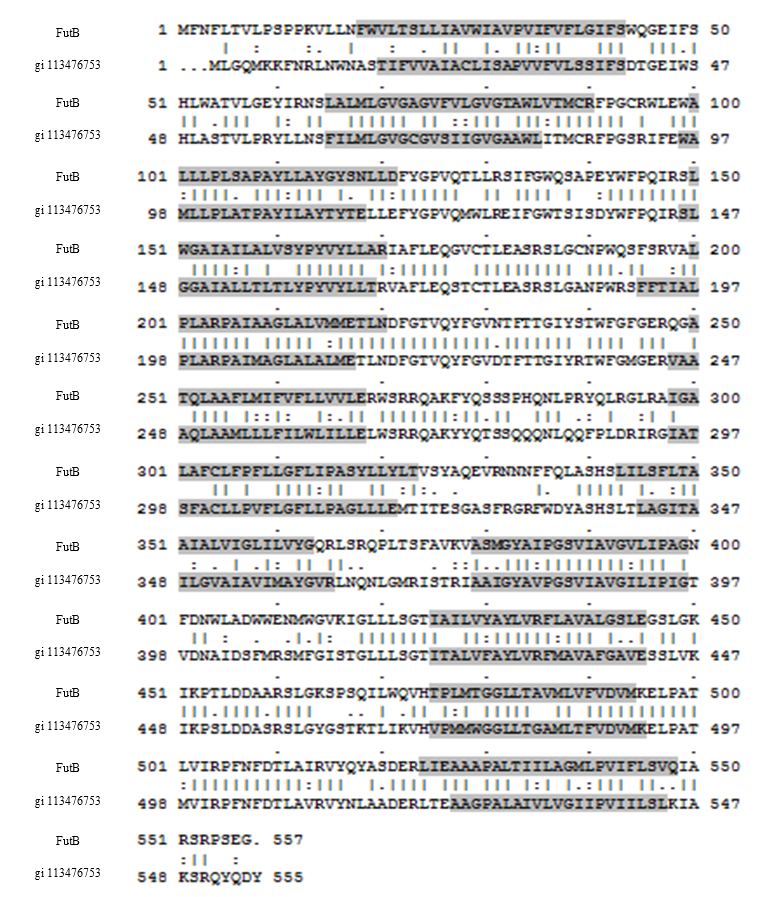


Fig. S3A. Alignment of the ferric iron porter, FutB, with gi113476753 yielding a comparison score of 305 S.D. with 67.5% similarity and 59.6% identity.


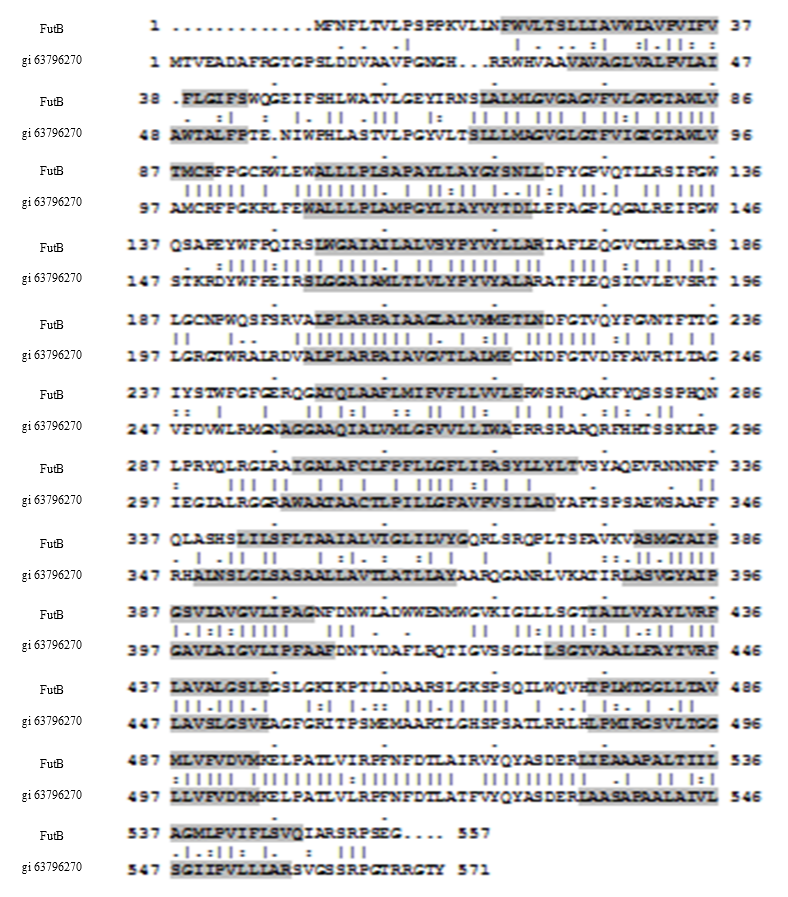


Fig. S3B. Alignment of the ferric iron porter, FutB, with gi163796270 yielded a comparison score of 188.3 S.D. with 57.7% similarity and 49.5% identity.


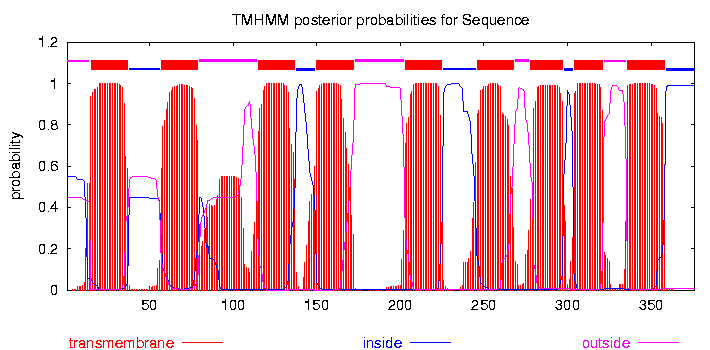


Fig. S4A. Hydropathy plot (TMHMM) of an RnsC (3.A.1.2.12) homologue, gi222147212.


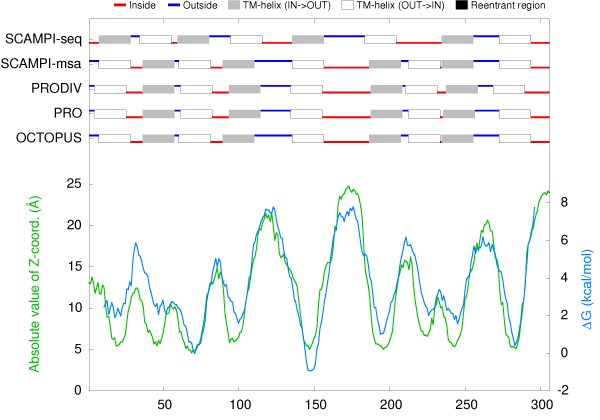


Fig. S4B. Topology prediction (TOPCONS) of an RnsC (3.A.1.2.12) homologue, gi218884703.

**
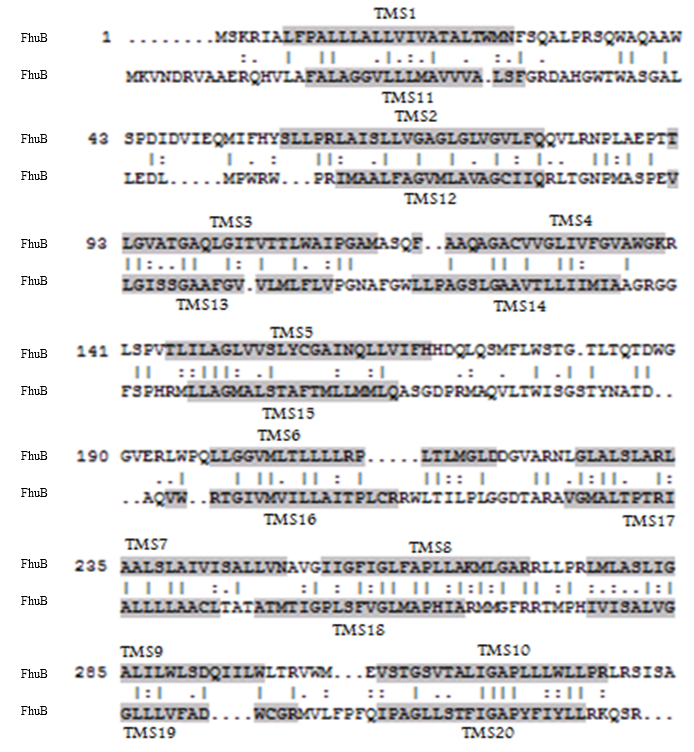
**

Fig. S5. TMSs 1-10 of FhuB aligning with TMSs 11-20, producing a comparison score of 33 S.D. (44.8% similarity and 31.5% identity).

**
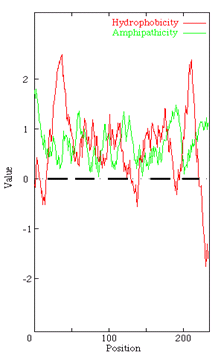
**

Fig. S6. Hydropathy plot (WHAT program) of HisM (3.A.1.3.1).

**
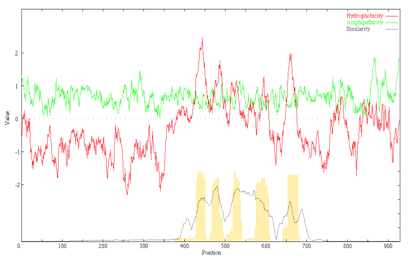
**

Fig. S7. Average hydropathy plot (top red line) using the AveHAS program for the top 100 non-redundant homologues of HisM. Top, red line, average hydropathy; top, green line, average amphipathicity; bottom, grey line, average similarity; bottom, yellow lines, an independent prediction of TMSs.

**
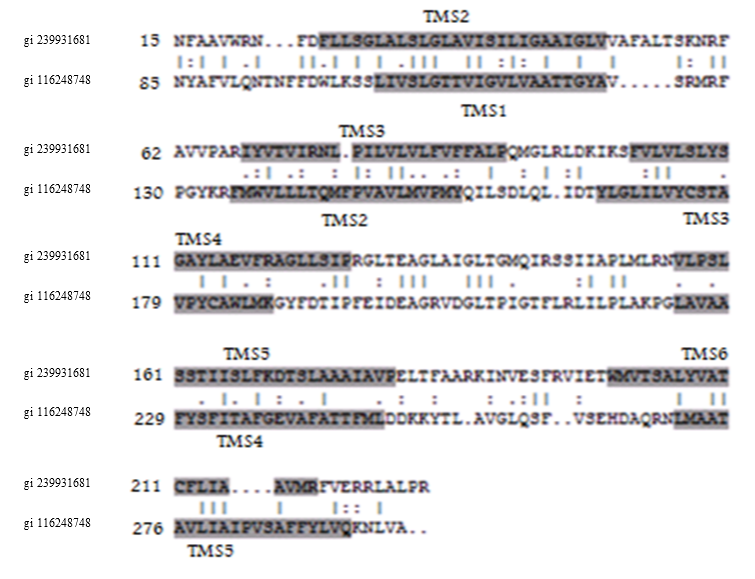
**

Fig. S8. TMSs 2-6 of a MalG homologue (gi239931681) aligned with TMSs 1-5 of a HisM homologue (gi116248748), resulting in a comparison score of 17.5 S.D. with 39.2% similarity and 27.9% identity.

**
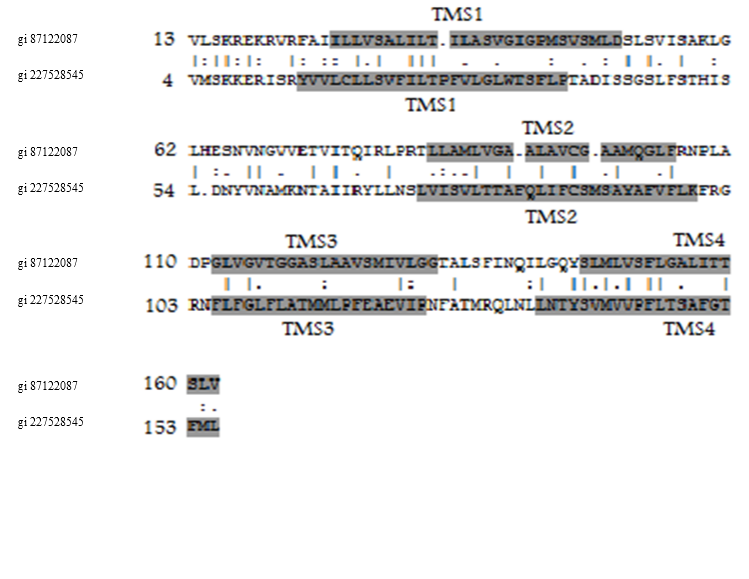
**

Fig. S9. GAP alignment between gi87122087 and gi227528545. The comparison score was 11.2 S.D. with 34.9% similarity and 24.8% identity.

**
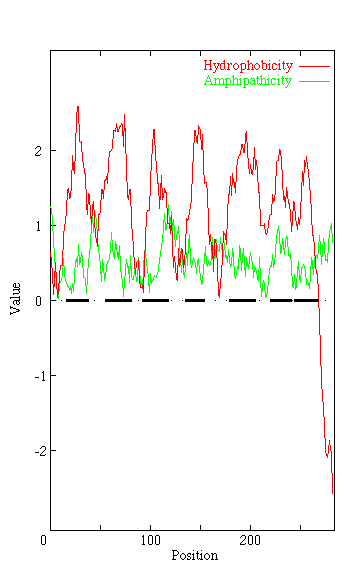
**

Fig. S10. Hydropathy plot of TogN (3.A.1.1.11), a putative seven TMS transporter.

.


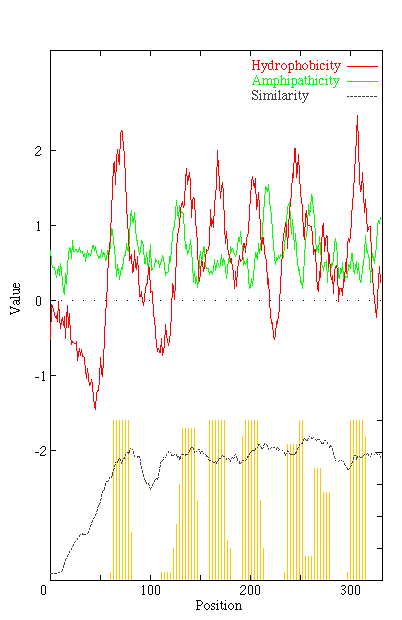


Fig. S11. Average hydropathy plot (AveHAS program) of the first 20 non-redundant homologues of TogN. Note the small size of peak 6. This may explain the discrepancy between the 6 and 7 TMS predictions (Table S2). See legend to figure S7 for further explanation.

**
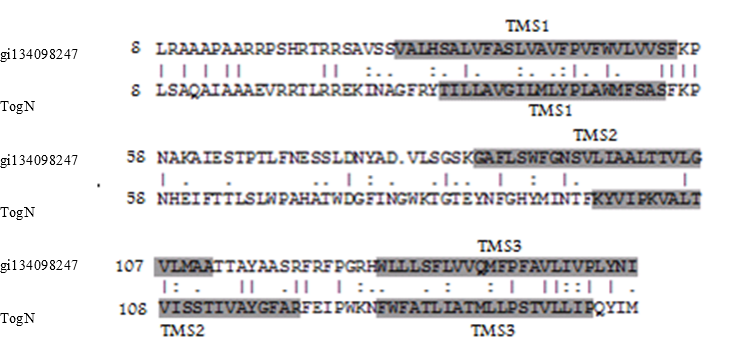
**

Fig S12. TMSs 1-3 of a MalG (3.A.1.1.1) homologue, gi134098247, aligning with TMSs 1-3 of TogN (3.A.1.1.11).

.

**
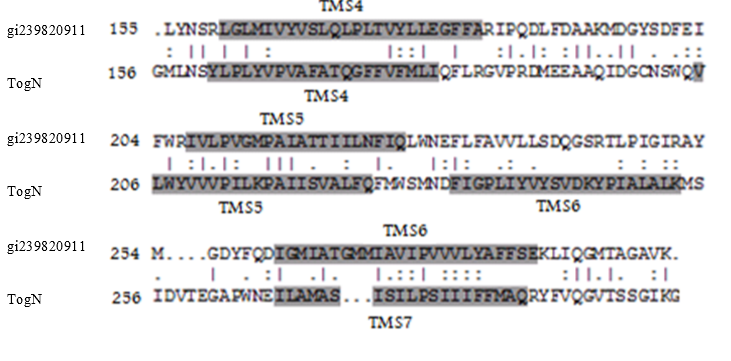
**

Fig. S13. Putative TMSs 4-6 of a MalG homologue, gi239820911, aligning with TMSs 4-7 of TogN (3.A.1.1.11).

.

**
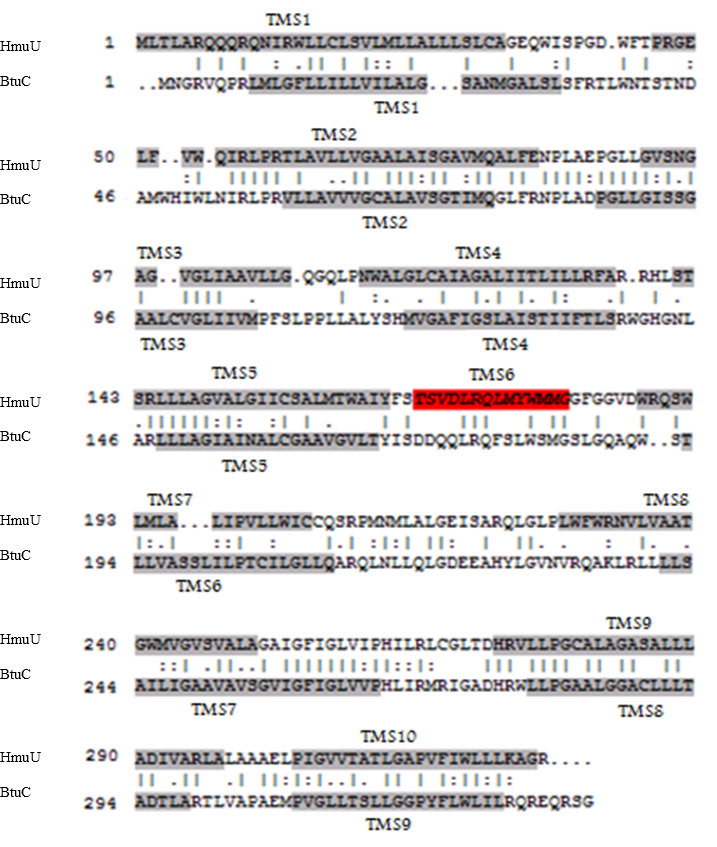
**

Fig. S14. Whole-protein alignment between putative nine TMS porter, HmuU, and an established ten TMS porter, BtuC, which was predicted to have 9TMSs. The comparison score was 55.5 S.D. with 52% similarity and 41.4% identity. The red segment is an unmatched TMS.

**
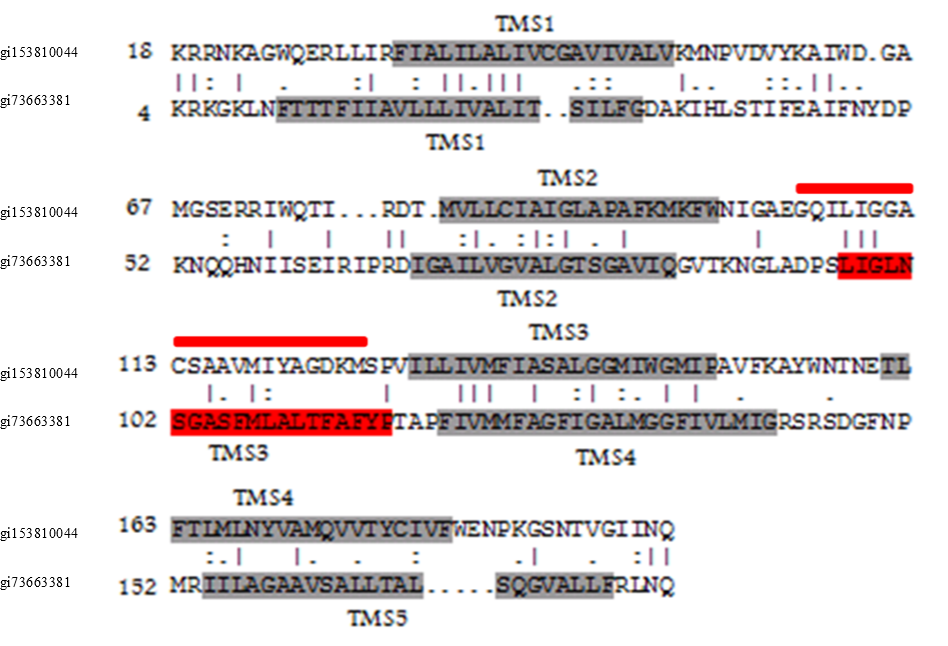
**

Fig. S15. GAP alignment between gi153810044 and gi73663381. The comparison score was 10.3 S.D. with 32.6% similarity and 22.7% identity. The red segment is a TMS matched using TOPCONS.

**
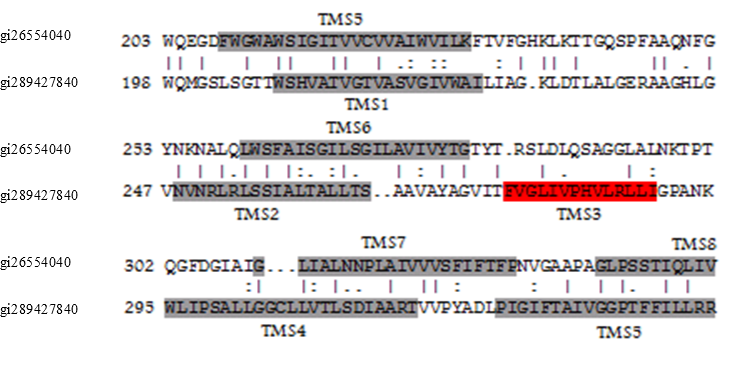
**

Fig. S16. GAP alignment between gi26554040 and gi289427840. The comparison score was 10.3 S.D. with 36.4% similarity and 27.9% identity. The red segment is an unmatched TMS.
